# *Drosophila* ADCK1 is critical for maintaining mitochondrial structures and functions in the muscle

**DOI:** 10.1371/journal.pgen.1008184

**Published:** 2019-05-24

**Authors:** Woongchang Yoon, Sun-Hong Hwang, Sang-Hee Lee, Jongkyeong Chung

**Affiliations:** 1 National Creative Research Initiatives Center for Energy Homeostasis Regulation, Institute of Molecular Biology and Genetics, Seoul National University, Gwanak-Gu, Seoul, Republic of Korea; 2 School of Biological Sciences, Seoul National University, Gwanak-Gu, Seoul, Republic of Korea; 3 Institute of Molecular Biology and Genetics, Seoul National University, Gwanak-Gu, Seoul, Republic of Korea; Stanford University School of Medicine, UNITED STATES

## Abstract

The function of AarF domain-containing kinase 1 (ADCK1) has not been thoroughly revealed. Here we identified that ADCK1 utilizes YME1-like 1 ATPase (YME1L1) to control optic atrophy 1 (OPA1) and inner membrane mitochondrial protein (IMMT) in regulating mitochondrial dynamics and cristae structure. We firstly observed that a serious developmental impairment occurred in *Drosophila ADCK1* (*dADCK1*) deletion mutant, resulting in premature death before adulthood. By using temperature sensitive ubiquitously expression driver *tub-Gal80*^*ts*^*/tub-Gal4* or muscle-specific expression driver *mhc-Gal4*, we observed severely defective locomotive activities and structural abnormality in the muscle along with increased mitochondrial fusion in the *dADCK1* knockdown flies. Moreover, decreased mitochondrial membrane potential, ATP production and survival rate along with increased ROS and apoptosis in the flies further demonstrated that the structural abnormalities of mitochondria induced by *dADCK1* knockdown led to their functional abnormalities. Consistent with the ADCK1 loss-of-function data in *Drosophila*, ADCK1 over-expression induced mitochondrial fission and clustering in addition to destruction of the cristae structure in *Drosophila* and mammalian cells. Interestingly, knockdown of *YME1L1* rescued the phenotypes of ADCK1 over-expression. Furthermore, genetic epistasis from fly genetics and mammalian cell biology experiments led us to discover the interactions among IMMT, OPA1 and ADCK1. Collectively, these results established a mitochondrial signaling pathway composed of ADCK1, YME1L1, OPA1 and IMMT, which has essential roles in maintaining mitochondrial morphologies and functions in the muscle.

## Author summary

Mitochondria function as energy producing factories in the cell, and thus the malfunctioning of mitochondria becomes the causes of many diseases. Especially in muscles that continuously require a vast amount of energy, dysfunction of mitochondria leads to abnormalities in muscles. Mitochondria maintain their homeostasis and recover from stresses induced by external stimuli through a dynamic process of continuous fusion and fission. Moreover, they constantly produce ATP through their wrinkled internal structure, called the cristae. We discovered that ADCK1 is important in maintaining these mitochondrial functions. In the fruit fly model, a severe developmental anomaly was observed in ADCK1 mutant, and inhibition of ADCK1 expression led to defects in locomotive activity, along with abnormalities in mitochondrial structure and functions in muscles. Interestingly, these anomalies in mitochondria were due to OPA1 and IMMT proteins that exist downstream of ADCK1, regulated by ADCK1 through a protease called YME1L1. These results provide better molecular understanding on how mitochondria contribute to degenerative diseases in the muscular system.

## Introduction

The ADCK family proteins have common structural signatures of protein serine/threonine or tyrosine kinase and the evolutionarily conserved AarF domain. Among the family proteins, ADCK3 (also known as COQ8A) is well-known for its functions related to production of coenzyme Q ubiquinone, which is an essential lipid-soluble carrier in the electron transport chain [1]. Similarly, there have been studies claiming that ADCK4 (also known as COQ8B) also participates in the production of coenzyme Q [2]. Interestingly, the mutated form of ADCK3 is reported to be the main cause of cerebellar ataxia with seizures as well as decrease in motility [3, 4]. Based on these isoform-specific functions, additional studies are needed to determine the functional roles of the other ADCK family proteins including ADCK1.

The cristae formations of the mitochondria increase the surface area of the mitochondrial inner membrane and plays important roles in maintaining ATP production [5]. Any damage to the structure of the cristae results in defective ATP production [6, 7]. In recent studies, it has been stated that the mitochondrial contact site and cristae organizing system (MICOS) complex is a key component in developing and maintaining the cristae junctional structure [8, 9]. The MICOS complex consists of many subunits and of these, IMMT (also known as MIC60) is recognized to be in the centre of the complex [10]. Although it is well-known that IMMT participates in the formation of the cristae structure, its mechanism was unknown. Defects in the MICOS complex result in many diseases such as amyotrophic lateral sclerosis (ALS), Charcot–Marie–Tooth (CMT) disease and optic atrophy [11–13]. In addition, there are many reports confirming the interaction between the MICOS complex and the sorting and assembly machinery (SAM) complex which anchors the beta-barrel proteins to the outer membrane of the mitochondria [14]. Therefore, if IMMT expression is suppressed, the assembly of the MICOS complex and the SAM complex is highly defected, and consequently the cristae structures are reported to nearly disappear, resulting in a concentric circle-shaped inner membrane [15]. In the cell, the functions of IMMT can be regulated by controlling its stability by an i-AAA type protease called YME1L1, located in the inner membrane of the mitochondria [10]. YME1L1 is thought to be associated with apoptosis and cell proliferation through maintenance of the cristae structure and the assembly of the respiratory chain subunits [16]. In addition, abnormal YME1L1 has been revealed to be the direct cause of optic atrophy 11 [17].

The mitochondrial fusion/fission was developed in the cell to adapt to the environmental changes and involves in many intracellular processes such as cellular stress responses, mitophagy, apoptosis, mitochondrial DNA stability, and respiratory capacity [18, 19]. Therefore, disruption in balance between fusion and fission hinders the cellular homeostasis and hence induces various abnormalities, such as neurodegenerative diseases [20]. Mitochondrial fusion and fission work antagonistic to each other, as the increase in fusion or fission leads to the decrease in the other [21]. The fusion is regulated by mitofusin1 (MFN1) and mitofusin2 (MFN2) for the outer membrane and by OPA1 for the inner membrane [22, 23]. Especially, OPA1 has a GTPase domain and exists in L-OPA1 form in the inner membrane and in S-OPA1 form in the intermembrane space, without the transmembrane domain. The central function of OPA1 is consistent with the activity of L-OPA1 in anchoring the inner membrane of mitochondria during mitochondrial fusion [24]. According to many recent findings, the importance of L-OPA1 in cristae formation has also been discovered [25–27]. Particularly, L-OPA1 was found to interact with the key subunit of the MICOS complex, IMMT, to maintain the cristae structure [28]. Although the function of S-OPA1 has not been well identified compared to L-OPA1, previous studies revealed that it is involved in the mitochondrial fission or assists L-OPA1 by forming an oligomer with L-OPA1 [24]. It has been discovered that a disturbance in the balanced state of L-OPA1 and S-OPA1 causes mitochondrial dysfunction and induces fragmentation, thereby affecting cells even to cell death [29]. The balance between L-OPA1 and S-OPA1 is regulated by changing the cleavage pattern of OPA1 by YME1L1 [30].

Our interest in this research was to identify the unknown functions of proteins residing in mitochondria. Through the discovery, we aimed to observe new functions and roles of the mitochondria in living organisms and to identify the causes and treatments of mitochondria-related diseases. Specifically, we predicted that ADCK1 would participate in a novel signal transduction cascade in the mitochondria. We unexpectedly found that ADCK1 plays a crucial role in mitochondrial dynamics and cristae formation through interacting with critical mitochondrial proteins, such as YME1L1, OPA1 and IMMT.

## Results

### *ADCK1* mutant flies show developmental defects

We aimed to find a novel regulatory protein existing in mitochondria or a pathogenic mechanism of mitochondrial diseases that was not well studied. Hence, we underwent a screening procedure to find a new mitochondrial regulator with an evolutionarily conserved catalytic domain. Among 159,743 human proteins, 35 final candidates were acquired through series of text mining processes (S1A Fig). These 35 candidates were again screened using RNAi-based fly genetics and *Drosophila* ADCK1 (dADCK1) was finally found to affect mitochondrial structures and dynamics.

The human ADCKs were encoded by a gene family of *ADCK1*, *ADCK2*, *ADCK3*, *ADCK4*, and *ADCK5*, whereas the fly has a family of *Adck* (*CG3608*), *CG32649*, and *CG7616*. According to homology database, *Drosophila Adck* is the homolog of human *ADCK1* (henceforth, *Drosophila* Adck is called dADCK1). CG32649 is the homolog of human *ADCK3* and *ADCK4*, and CG7616 is the homolog of human *ADCK5*. The fruit fly homolog of human *ADCK2* has not been found.

**Fig 1:**
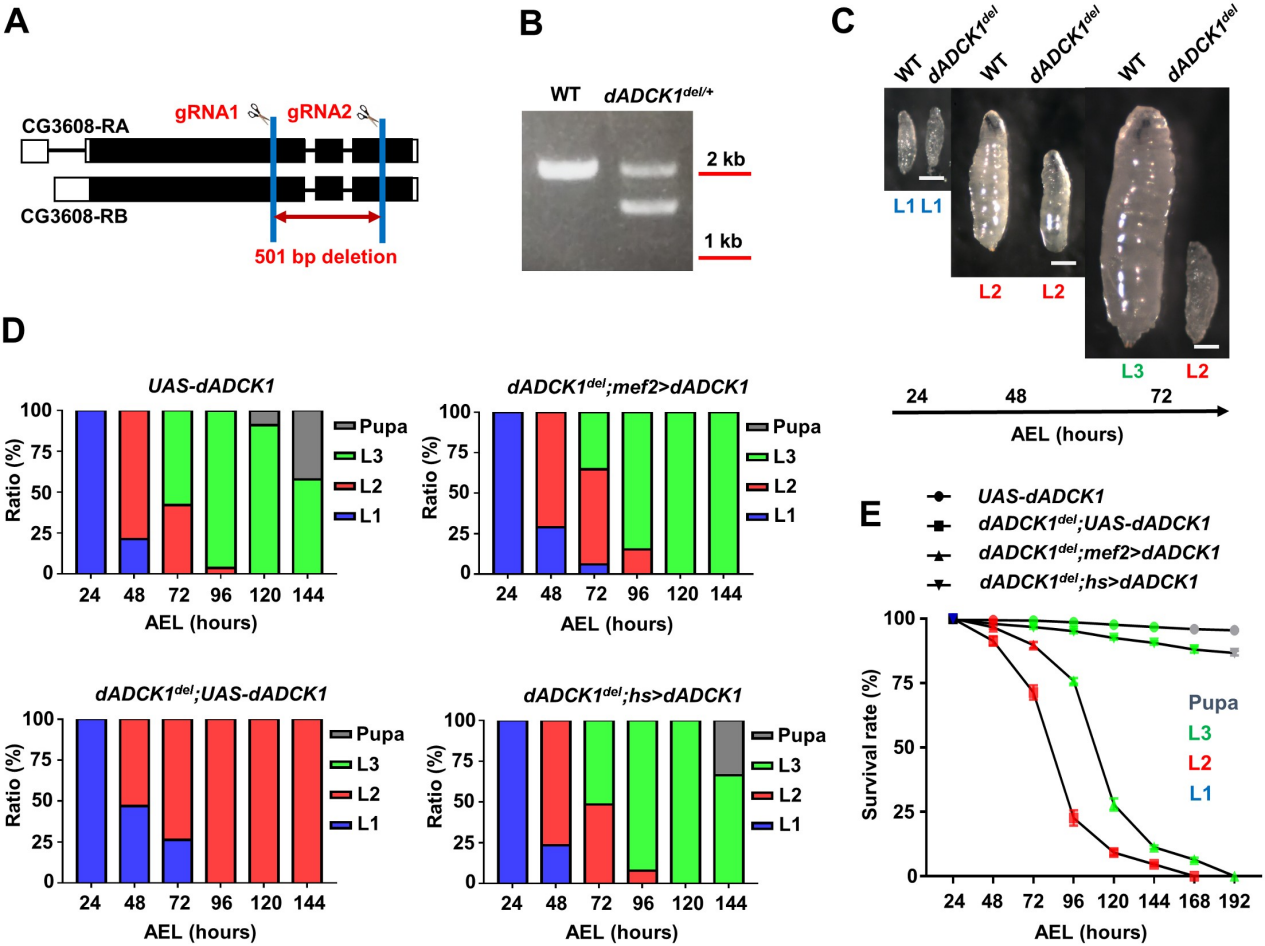
*ADCK1* mutant flies show development defects. (A) A scheme to generate a *dADCK1* deletion allele (*dADCK1*^*del*^) by CRISPR/Cas9. (B) A DNA gel electrophoresis result for the genomic DNA of wild type (WT) and heterozygous *dADCK1* mutant allele (*dADCK1*^*del/+*^). (C) Comparison of the body size and developmental stages between WT (left) and *dADCK1*^*del*^ (right) flies. WT flies were first instar (L1), second instar (L2), and third instar (L3) larvae at 24, 48, and 72 hours after egg laying (AEL), respectively. *dADCK1*^*del*^ flies in these photos were first instar and second instar larvae, collected at the same time points. Scale bars, 0.1 mm. (D) Developmental progression of the flies with indicated genotypes. n = 375~403. (E) Comparison of the viability of the flies with indicated genotypes. n = 375~403. Statistical significance was analyzed by log-rank (Mantel-Cox) test. p<0.0001. Color-coded symbols indicate specific developmental stages.

In this study, we have generated a loss-of-function mutant of *dADCK1* in order to understand the in vivo function of *ADCK1*. We produced the mutant by deleting the *dADCK1* gene from the end of the 1^st^ exon to the middle of the 3^rd^ exon using two guide RNAs by CRISPR/Cas9 system (Fig 1A) [31]. As a result, we created a *dADCK1* deletion mutant (Fig 1B) without 501 base pairs of the evolutionarily very-well conserved amino acids positioned at 349–473 (S1B Fig). The *dADCK1* homozygous mutant flies showed defects in development. Compared to control larvae, the homozygote mutant showed a smaller body size in the second instar larva stage (Fig 1C). In addition, the mutant flies required an abnormally longer time of transition from the first instar stage to the second instar stage and could not proceed into the third instar stage. However, when exogenous dADCK1 was over-expressed in the homozygote mutant flies by muscle-specific *mef2-Gal4* driver, the mutant larvae were partially rescued and developed into the third instar stage (Fig 1D and 1E). Furthermore, when exogenous dADCK1 was over-expressed by ubiquitous *hs-Gal4* driver, the mutants developed normally into the adult stage (Fig 1D and 1E). We also observed that the survival rates of the *dADCK1* homozygote mutants were notably lower than control flies and could not survive longer than 7 days after egg laying (AEL). Yet, the short survival rate of the *dADCK1* homozygote mutant flies was rescued by dADCK1 over-expression by *hs-Gal4* driver (Fig 1E). Through these observations, we concluded that dADCK1 is critical for normal development of *Drosophila*.

### *ADCK1* knockdown causes abnormality in the muscle

**Fig 2:**
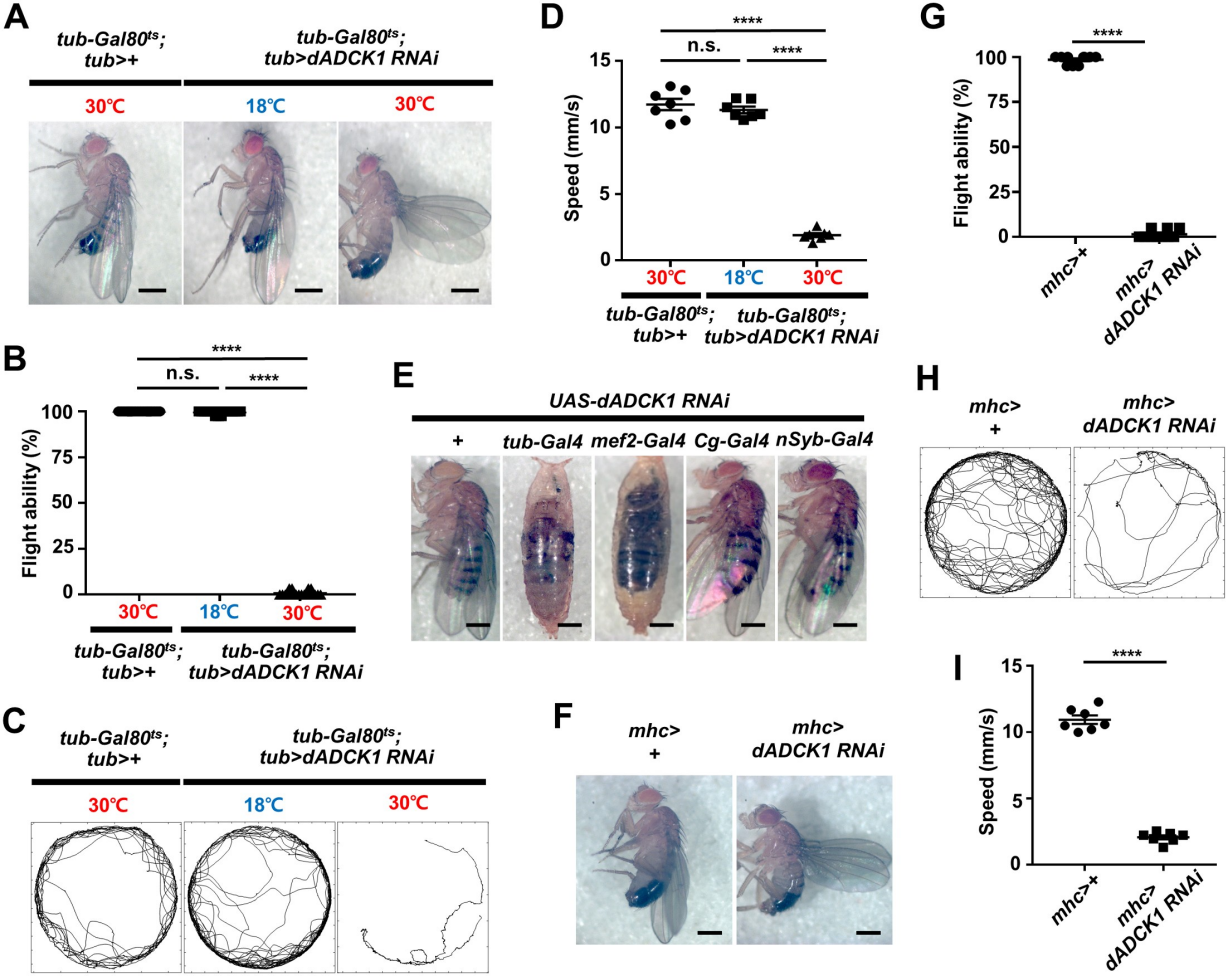
*ADCK1* knockdown causes abnormality in locomotive activity. (A) Whole body images of the adult flies with indicated genotypes raised at noted temperatures. Scale bars, 0.5 mm. (B) Comparison of the flight ability for the flies with indicated genotypes. n = 20. ****, p<0.0001; n.s., not significant by unpaired t-test. (C) Movement trajectories of the adult flies with indicated genotypes and raised temperatures. (D) Comparison of the means of walking speed for the flies with indicated genotypes, n = 7. ****, p<0.0001; n.s., not significant by unpaired t-test. (E) Whole body images of the flies with indicated genotypes. *dADCK1* knockdown using ubiquitous *tub-Gal4* driver, muscle-specific *mef2-Gal4* driver, fat body-specific *Cg-Gal4* driver, and neuron-specific *nSyb-Gal4* driver. Scale bars, 0.5 mm. (F) Whole body images of the adult flies with indicated genotypes. Scale bars, 0.5 mm. (G) Comparison of the flight ability for the flies with indicated genotypes. n = 10. ****, p<0.0001 by unpaired t-test. (H) Movement trajectories of the adult flies with indicated genotypes. (I) Comparison of the means of walking speed for the flies with indicated genotypes, n = 7. ****, p<0.0001 by unpaired t-test.

Due to the early lethal phenotype of the *dADCK1* homozygote mutant (Fig 1E), we studied the function of dADCK1 using RNA interference knockdown by UAS-Gal4 system. We first tested *tub-Gal4* that is expressed in the entire body of *UAS-dADCK1 RNAi* flies, and we were again able to confirm that the flies die without dADCK1 during development (S2A Fig). Thus, we started to use the *Gal80*^*ts*^ system which represses the expression of Gal4 driver depending on the culture temperature in order to observe the effect of *dADCK1* knockdown in an adult fly [32]. In our experiment, we grew our flies at low temperature of 18°C and allowed them to grow into adulthood by repressing *tub-Gal4* driver. Thereafter, we performed *dADCK1* knockdown on adult flies by temporarily increasing the temperature to 30°C for 7 days to induce *tub-Gal4*. As a result, we were able to confirm that if the expression of *dADCK1* was repressed by inducing *dADCK1* RNAi, the flies showed defective ‘held-up’ wing position phenotypes (Fig 2A) and flight disability (Fig 2B). Next, the behavior of the flies was observed with the video tracking system and their locomotive activities were analyzed [33]. It was notable that *dADCK1* RNAi flies at 30°C displayed an obvious decline in the movement and the walking speed analyses than either *dADCK1* RNAi flies at 18°C or the control flies at 30°C (Fig 2C and 2D).

When *dADCK1* was knocked down using ubiquitous *tub-Gal4* driver, muscle-selective *mef2-Gal4* driver, adipocyte-selective *Cg-Gal4* driver, and neuron-specific *nSyb-Gal4* driver, the flies expressing *dADCK1* RNAi in adipocytes and neurons were able to successfully grow into adulthood without significant defects (S3A, S3B and S3C Fig). However, the flies expressing *dADCK1* RNAi by ubiquitous *tub-Gal4* and muscle-selective *mef2-Gal4* drivers showed lethal phenotypes (Fig 2E).

The *mhc-Gal4* driver selectively expresses target genes in muscle tissues and is less strongly expressed than *mef2-Gal4* driver, which often allows the transgenic flies to grow into adulthood. Hence, using *mhc-Gal4* driver, we were able to confirm that *dADCK1* knockdown flies (S2B Fig) had defective wing phenotypes (Fig 2F), flight ability (Fig 2G) and locomotive activity (Fig 2H and 2I and S2C and S2D Fig), compared to the control flies. These results strongly supported the importance of dADCK1 in the muscle.

### *ADCK1* knockdown causes mitochondrial abnormality in the muscle

**Fig 3:**
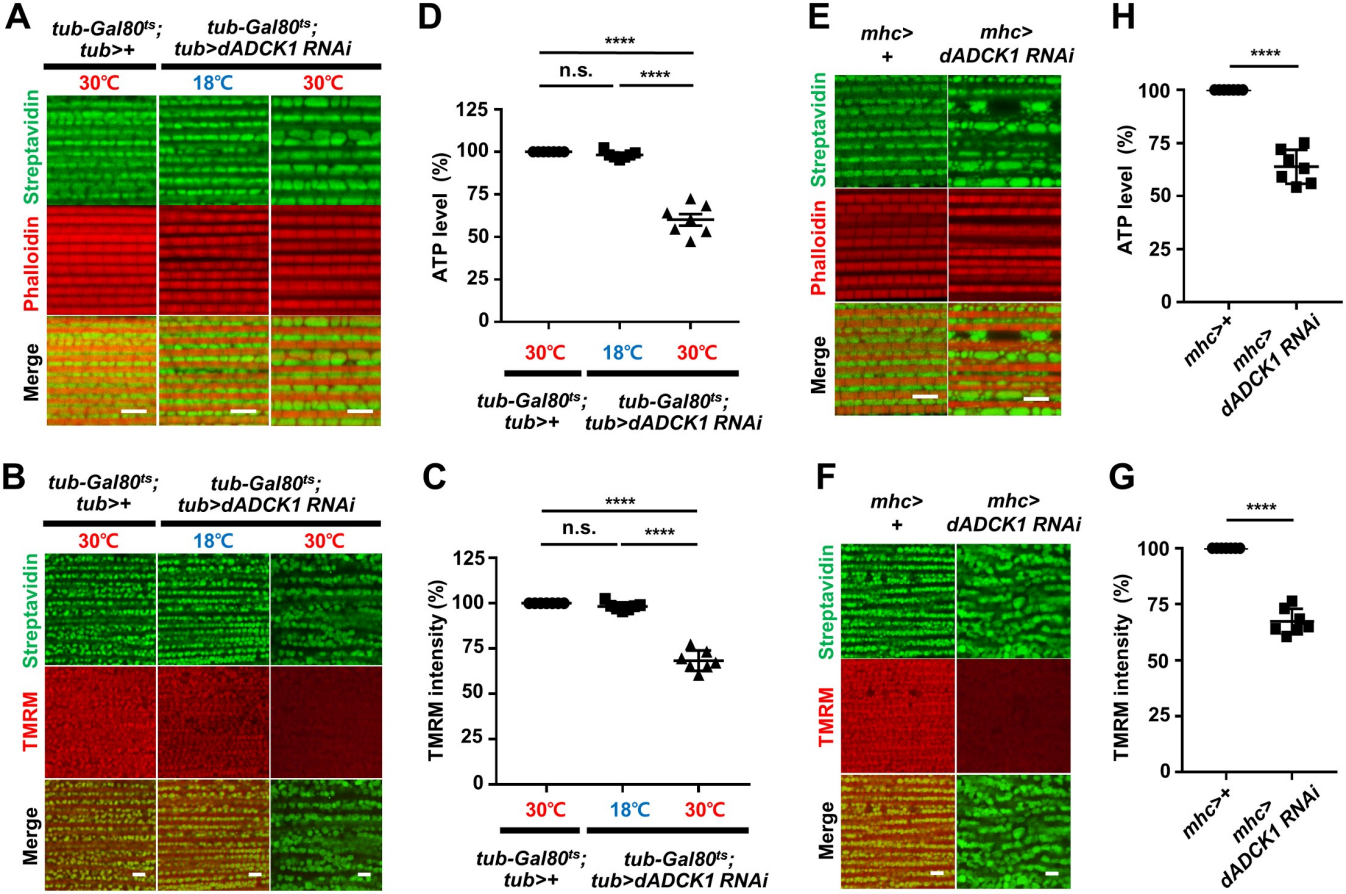
*ADCK1* knockdown causes mitochondrial abnormality in the muscle. (A) Fluorescent confocal microscopy images of the adult fly thoraces with indicated genotypes and raised temperatures. Streptavidin (green) and phalloidin (red) staining were used to mark mitochondria and muscle actin fibers, respectively. 1,500× magnification. Scale bars, 5 μm. (B) Fluorescent confocal microscopy images of the adult fly thoraces with indicated genotypes and raised temperatures. Mitochondrial membrane potentials were measured by TMRM staining (red). 1,000× magnification. Scale bars, 5 μm. (C) Comparison of the mitochondrial membrane potentials with indicated genotypes and raised temperatures. n = 7. ****, p<0.0001; n.s., not significant by unpaired t-test. (D) Comparison of ATP levels in the adult fly thoraces with indicated genotypes. n = 7. ****, p<0.0001; n.s., not significant by unpaired t-test. (E) Fluorescent confocal microscopy images of the adult fly thoraces with indicated genotypes. 1,500× magnification. Scale bars, 5 μm. (F) Fluorescent confocal microscopy images of the adult thoraces with indicated genotypes. 1,000× magnification. Scale bars, 5 μm. (G) Comparison of the mitochondrial membrane potentials with indicated genotypes. n = 7. ****, p<0.0001 by unpaired t-test. (H) Comparison of ATP levels in the adult fly thoraces with indicated genotypes. n = 7. ****, p<0.0001 by unpaired t-test.

From previous experiments, we were able to establish that decrease in the locomotive activity of the *dADCK1* loss-of-function flies was due to muscle abnormalities (Fig 2E–2I). To further investigate the cause of the abnormalities, we dissected the adult fly thorax and stained with streptavidin and phalloidin to mark mitochondria and muscle actin fibers, respectively. We used temperature-dependent *tub-Gal80*^*ts*^ and ubiquitously-expressing *tub-Gal4* driver to selectively induce *dADCK1* RNAi at 30°C, and their thoraces were dissected and observed. Interestingly, the thoracic muscles were abnormally oriented and showed deformed mitochondrial morphologies with increased mitochondrial fusion in *dADCK1* RNAi flies at 30°C compared to the *dADCK1* RNAi flies at 18°C or the control flies at 30°C (Fig 3A). In order to determine if there are dysfunctional mitochondria amongst the abnormally structured mitochondria, we measured the mitochondrial membrane potential in the thorax muscle of the adult flies using tetramethylrhodamine methyl ester (TMRM) staining. We observed that the *dADCK1* RNAi flies at 30°C had decreased mitochondrial membrane potentials compared to those of the *dADCK1* RNAi flies at 18°C or the control flies at 30°C (Fig 3B and 3C). We also measured the amount of ATP in the thorax muscle of these flies. We discovered that the thorax muscle of the *dADCK1* RNAi flies at 30°C had decreased amount of ATP compared to the *dADCK1* RNAi flies at 18°C or the control flies at 30°C (Fig 3D).

By performing *dADCK1* knockdown using muscle-specific *mhc-Gal4* driver as well, we were able to confirm the *dADCK1* knockdown flies had abnormal muscle structure and increased mitochondria fusion compared to the control flies (Fig 3E). In the same experiments, we observed decreased TMRM staining in the *dADCK1* knockdown flies (Fig 3F and 3G). In addition, the amount of ATP in the thorax muscle of the *dADCK1* knockdown flies was decreased compared to the control flies (Fig 3H). Based on these results, we concluded that *dADCK1* knockdown induces mitochondrial fusion and dysfunction in muscle cells.

### *ADCK1* knockdown increases ROS level and apoptosis

**Fig 4:**
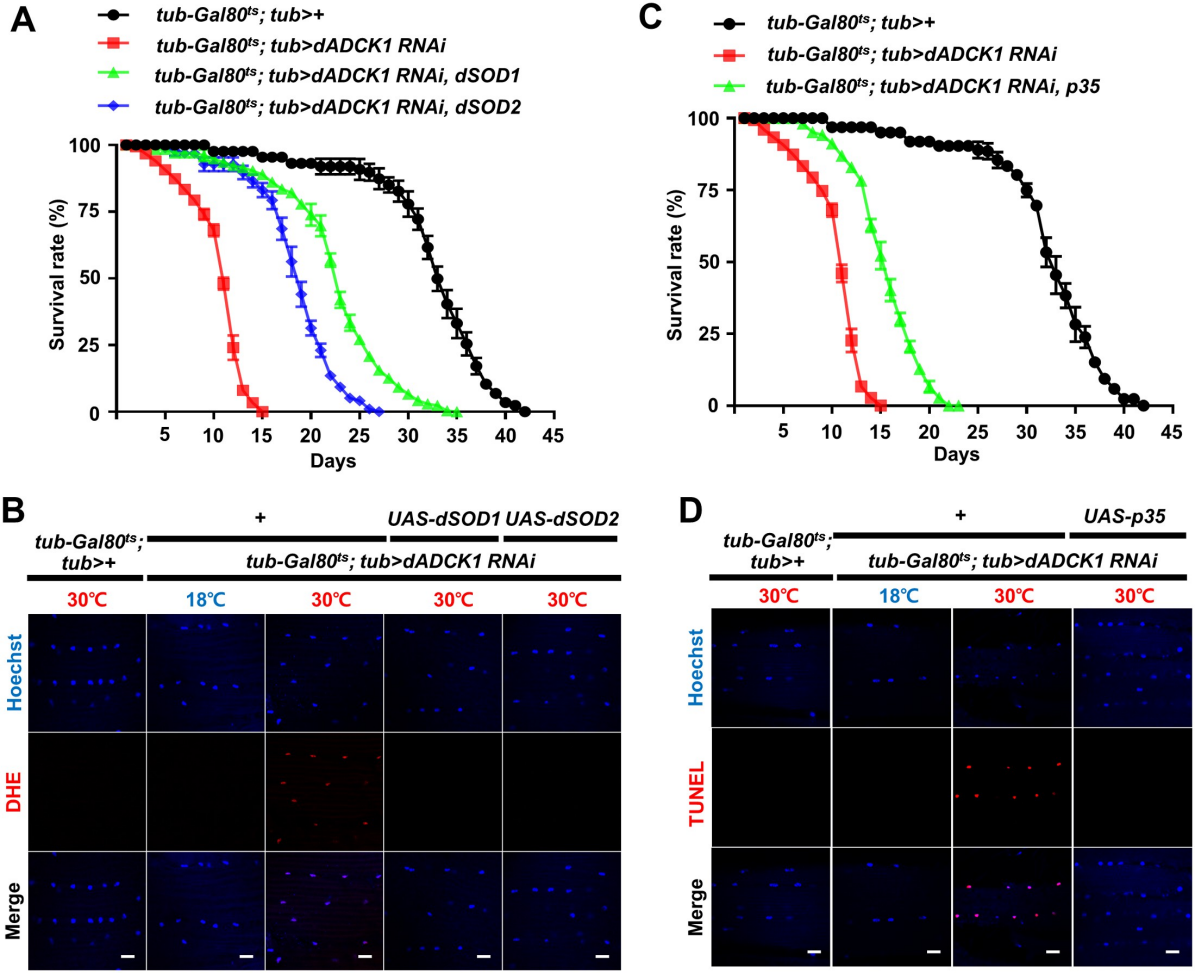
*ADCK1* knockdown increases ROS level and apoptosis. (A) Comparison of the survival rate for the flies with indicated genotypes. n = 80. Statistical significance was analyzed by log-rank (Mantel-Cox) test. p<0.0001. (B) Fluorescent confocal microscopy images of the adult fly thoraces with indicated genotypes and raised temperatures. Hoechst (blue) was used to mark the nuclei and DHE staining (red) was used to measure ROS level. Scale bars, 5 μm. (C) Comparison of the survival rate of the flies with indicated genotypes. n = 60. Statistical significance was analyzed by log-rank (Mantel-Cox) test. p<0.0001. (D) Fluorescent confocal microscopy images of the adult fly thoraces with indicated genotypes and raised temperatures. Hoechst (blue) was used to mark the nuclei and TUNEL staining (red) was used to detect apoptosis. Scale bars, 5 μm.

During ATP production in the mitochondria, reactive oxygen species (ROS) can be produced but the level of ROS production is well-controlled under normal circumstances [34]. Thus, mitochondrial damage affects both ATP and ROS production in the cell [35]. We raised adult flies at 30°C and observed the survival rate in order to determine if there were any changes in ROS level due to defective mitochondria. We confirmed that the *dADCK1* knockdown flies died earlier than the control flies (Fig 4A). When the ROS scavenger *Drosophila* superoxide dismutase 1 (*dSOD1*) and 2 (*dSOD2*) [36] were over-expressed in the *dADCK1* knockdown flies, the survival rate was partially recovered (Fig 4A). To confirm the changes in ROS level due to *dADCK1* knockdown, we performed dihydroethidium (DHE) staining in the thorax muscle. As a result, *dADCK1* knockdown at 30°C showed increased DHE staining compared to the *dADCK1* RNAi flies at 18°C or the control flies at 30°C, and the increased ROS level of *dADCK1* knockdown flies at 30°C was highly suppressed by over-expression of dSOD1 or dSOD2 (Fig 4B).

Next we examined the increased apoptosis upon *dADCK1* knockdown by observing the survival rate of the flies at 30°C. The *dADCK1* knockdown flies died faster, and hence showed decreased survival rates than the control flies. However, when p35, the baculovirus inhibitor for caspases [37], was expressed in the *dADCK1* knockdown flies, the characteristically low survival rate of *dADCK1* knockdown flies was partially recovered (Fig 4C). Unlike the *dADCK1* RNAi flies at 18°C or the control flies at 30°C, the *dADCK1* RNAi flies at 30°C showed strong TUNEL signals, confirming an increase in apoptosis, which was suppressed by p35 over-expression (Fig 4D). Overall, we were able to demonstrate that *dADCK1* knockdown increases ROS as well as apoptosis in the muscle.

### ADCK1 controls mitochondrial structures

**Fig 5:**
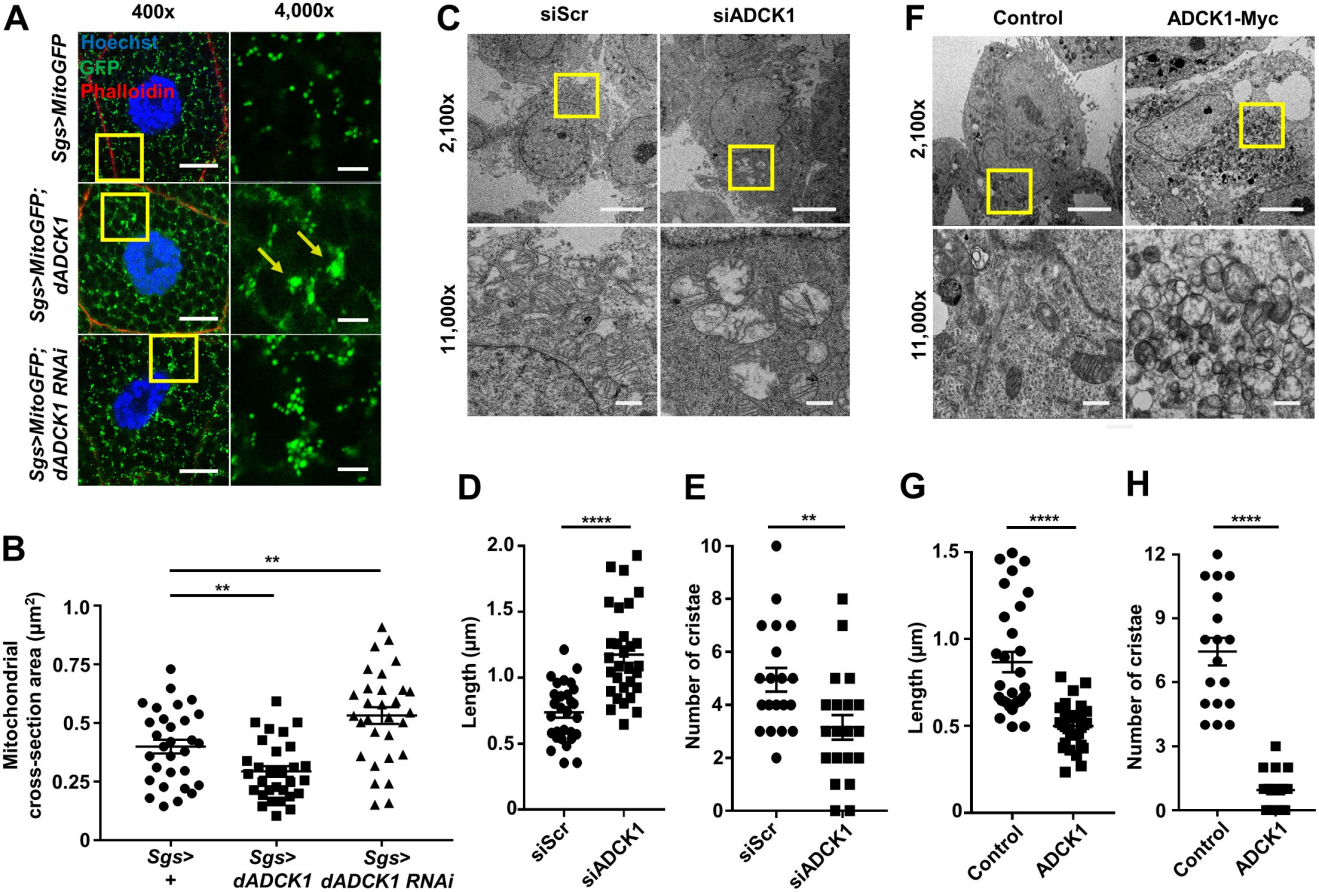
ADCK1 controls mitochondrial structures. (A) Fluorescent confocal microscopy images for the salivary glands of the third instar larvae with indicated genotypes. MitoGFP (green) shows mitochondria and phalloidin (red) staining indicates the cell boundary. Hoechst (blue) was used for nuclei staining. Scale bars, 20 μm (400×) and 5 μm (4,000×). Boxed areas were enlarged as shown on the right panels. Arrows direct clustered mitochondria. (B) Comparison of the mitochondrial cross-section area in the salivary glands of the third instar larvae with indicated genotypes. n = 30. **, p<0.01 by unpaired t-test. (C) TEM images of HeLa cells transfected with scramble siRNA (Scr) or siADCK1 as indicated. The boxed areas were enlarged in the bottom panels. Scale bars, 5 μm (2,100×, top) and 500 nm (11,000×, bottom). (D) Comparison of the mitochondrial length in HeLa cells transfected with siScr or siADCK1. n = 30. ****, p<0.0001 by unpaired t-test. (E) Comparison of the number of mitochondrial cristae in HeLa cells transfected with siScr or siADCK1. n = 20. **, p<0.01 by unpaired t-test. (F) TEM images of HeLa cells expressing empty vector or ADCK1-Myc as indicated. Scale bars, 5 μm (2,100×, top) and 500 nm (11,000×, bottom). The boxed areas were enlarged in the bottom panels. (G) Comparison of the mitochondrial length in controls and HeLa cells transfected with ADCK1. n = 30. ****, p<0.0001 by unpaired t-test. (H) Comparison of the number of mitochondrial cristae in controls and HeLa cells transfected with ADCK1. n = 18. ****, p<0.0001 by unpaired t-test.

In previous experiments, we examined the mitochondrial morphologies in the muscle of the *dADCK1* knockdown flies. As a result, we discovered that *dADCK1* knockdown facilitates mitochondrial fusion (Fig 3A and 3E). To further investigate this phenomenon, we performed *dADCK1* knockdown or over-expression using *Sgs-Gal4* driver in the salivary gland of *Drosophila* to better observe mitochondrial fusion and fission. Mitochondria in the salivary gland are spread out over a wide area in the cell, which makes us possible to examine typical mitochondrial morphologies from various angles. In consequence, mitochondrial fusion was increased in the cells of salivary glands from the *dADCK1* knockdown flies (Fig 5A and 5B). In contrast, increased mitochondrial fission and clustering of mitochondria was observed in the salivary gland of the *dADCK1* over-expression flies (Fig 5A and 5B).

To investigate whether these mitochondrial morphologies in *Drosophila* can be observed also in human cells, siRNA was applied to knockdown *ADCK1* in HeLa cells (S4A Fig) and observed through transmission electron microscopy (TEM) (Fig 5C). As a result, the mitochondrial length was increased and the number of mitochondrial cristae was decreased by *ADCK1* knockdown (Fig 5C–5E), suggesting the elevated mitochondrial fusion and cristae abnormality in *ADCK1* knockdown cells. In contrast with the knockdown results, when ADCK1 was over-expressed, mitochondrial abnormality was also obvious in TEM analyses (Fig 5F). The cells with ADCK1 over-expression resulted in mitochondrial fission with reduced mitochondrial length (Fig 5G). Moreover, ADCK1 over-expression caused severe destruction in cristae structure and thus resulted in abnormal circular shapes, clearly different from that of the control (Fig 5H and S4B Fig). Lastly, the mitochondrial clustering phenotype was observed once again (S4C Fig). These experimental observations led us to conclude the regulatory role of ADCK1 in mitochondrial fusion/fission and its critical role in formation of mitochondrial cristae structures.

### YME1L1 is a critical target of ADCK1

Through earlier experiments, we demonstrated that the decrease in locomotive activity may be induced by mitochondrial malfunction from *ADCK1* knockdown (Figs 2 and 3). Particularly, mitochondrial anomalies in structure and function may result from affecting proteins related to mitochondrial fusion/fission or formation of cristae structure. Hence, we have conducted a screening experiment to find genes that alters the phenotypes of *dADCK1* over-expression. First, we extracted 1,730 protein-coding genes existing in human mitochondria from the Gene Ontology (GO) database. From these listed genes, we obtained genes related to the phenotypes of *dADCK1* over-expression or knockdown, such as mitochondrial fusion/fission, cristae structure maintenance, ROS production and apoptosis. Finally, we established 101 candidate genes that were orthologous to the genes of fruit fly (S5 Fig). The RNAi lines of these final candidate genes were acquired from *Drosophila* stock centers to cross with the muscle-specific *dADCK1* over-expression fly (*mef2>dADCK1*). The descendant flies were then examined if they could rescue the developmental lethality seen characteristically in the *dADCK1* over-expression fly. After this genetic screen, the RNAi lines that rescued the developmental defects were again crossed with another muscle-specific *dADCK1* over-expression fly (*mhc>dADCK1*). The descendant flies were once again observed if they could alter the mitochondrial fission or clustering phenotype seen in the adult thorax of the *dADCK1* over-expression fly. As a result, we found out that dYME1L1 could change the phenotypes of the *dADCK1* over-expression fly among other candidates.

**Fig 6:**
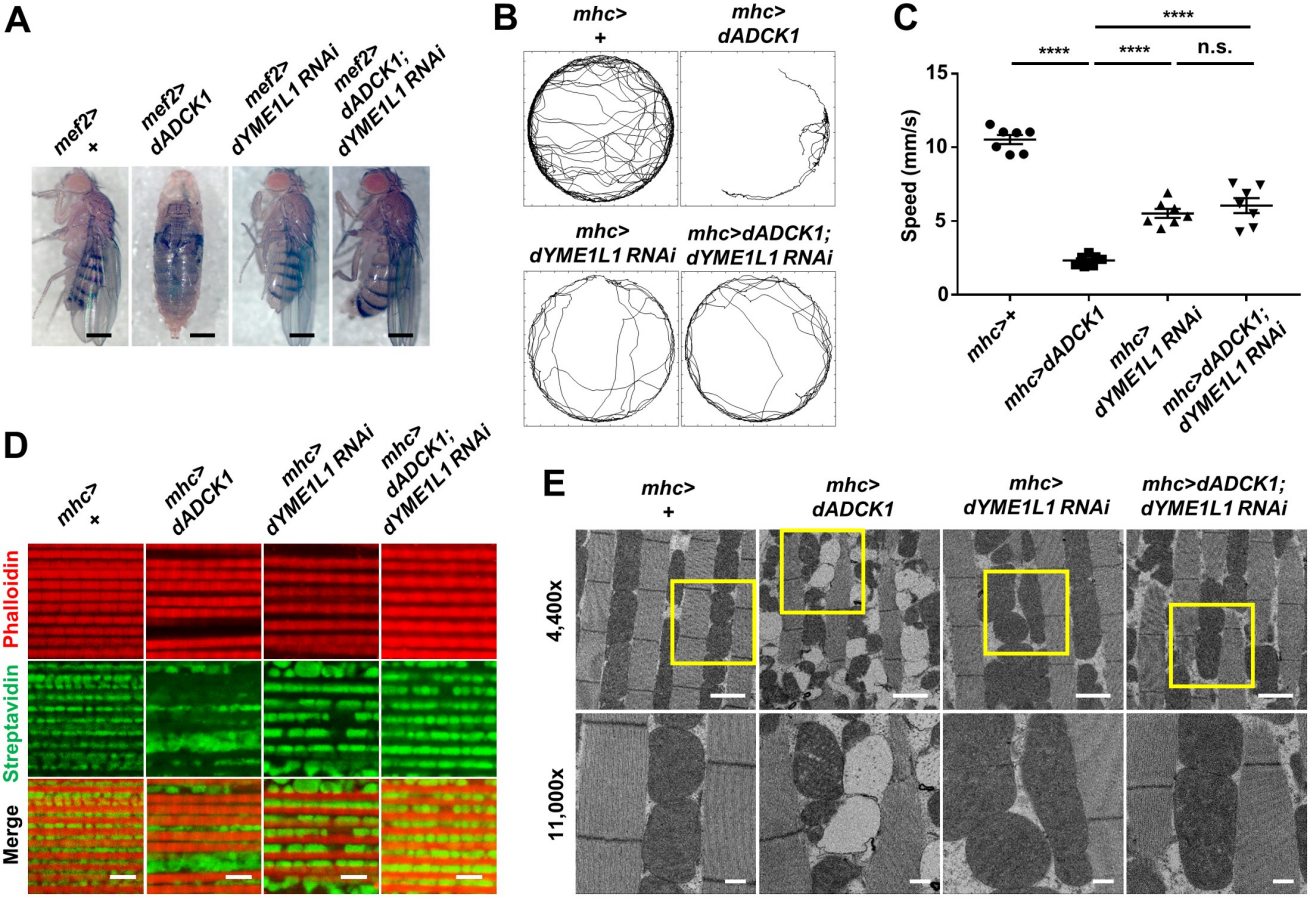
YME1L1 is a target of ADCK1. (A) Whole body images of the flies with indicated genotypes. Scale bars, 0.5 mm. (B) Movement trajectories of the adult flies with indicated genotypes. (C) Comparison of the means of walking speed for the flies with indicated genotypes, n = 7. ****, p<0.0001; n.s., not significant by unpaired t-test. (D) Fluorescent confocal microscopy images of the adult fly thoraces with indicated genotypes. Streptavidin (green) and phalloidin (red) staining used to mark mitochondria and muscle actin fibers, respectively. Scale bars, 5 μm. (E) TEM images of adult fly thoraces with indicated genotypes. Scale bars, 2 μm (4,400×, top) and 500 nm (11,000×, bottom). The boxed areas were enlarged in the bottom panels.

We previously showed that the *dADCK1* over-expression flies showed lethal phenotype in the pupa stage. Interestingly, the flies over-expressing *dADCK1* with simultaneous *dYME1L1* knockdown successfully survived into adulthood (Fig 6A), implicating that dYME1L1 is a target of dADCK1. We further wanted to know if such relationship between dADCK1 and dYME1L1 is applicable to the locomotive activity. By observing walking trajectory and speed, we confirmed that the locomotive activity of the *dADCK1* over-expressing adult flies dropped significantly compared to the controls. In this circumstance, the locomotive activity of the flies over-expressing *dADCK1* with simultaneous *dYME1L1* knockdown was similar to that of the *dYME1L1* knockdown flies (Fig 6B and 6C), suggesting that dADCK1 exists upstream of dYME1L1.

Next, we dissected the thoracic muscles of the flies and found that the mitochondrial defects of the *dADCK1* over-expressing flies were rescued by concurrent knockdown of *dYME1L1* (Fig 6D). We also observed the same thoracic muscles through TEM (Fig 6E). In the *dADCK1* over-expression fly, the mitochondria were frequently divided into normal (electron dense) and damaged (empty) parts, and the cristae structure was severely destroyed (Fig 6E). Moreover, we observed increased fission and clustering in the mitochondria of the fly (Fig 6E). Excitingly, these defective mitochondrial structures in the *dADCK1* over-expression fly were recovered close to normal by *dYME1L1* knockdown (Fig 6E), further confirming that dYME1L1 is a crucial target of dADCK1.

### IMMT functions downstream of ADCK1

The defects in mitochondrial cristae structure due to the over-expression of ADCK1 was shown in the previous experiments (Fig 5F). Hence, by analyzing interactions among the proteins under ADCK family, we aimed to discover proteins maintaining mitochondrial cristae structure while also interacting with ADCK1 and YME1L1. When human ADCK family proteins were phylogenetically analyzed using multiple sequence alignments, we were able to classify them into 3 groups of evolutionarily closer genes: *ADCK1* and *ADCK5*, *ADCK3* and *ADCK4*, and *ADCK2*. Notably, in *Drosophila*, *dADCK1* and *CG7616* (henceforth, *dADCK5*) were evolutionarily closer than *CG32649* (henceforth, *dADCK3/4*), which was consistent with the analysis of the human ADCK proteins (S6A Fig). When the human ADCK family genes were over-expressed, we observed that only ADCK1 and ADCK5 showed an increase in mitochondrial fission and clustering (S6B Fig). Similarly, when *Drosophila* ADCK family proteins were over-expressed in human cells, only dADCK1 and dADCK5 exhibited abnormal mitochondria phenotypes (S6C Fig). Therefore, we created a protein-protein interaction (PPI) network of the ADCK family proteins to find a common protein that interacts with ADCK1 and ADCK5. This led us to discover that ADCK1 and ADCK5 selectively interact with IMMT (S7A Fig). Next, we tried to find a protein that interacts with ADCK1 and IMMT as well. As a result, we discovered that YME1 interacts with both MCP2, the orthologue of ADCK1, and MIC60, the orthologue of IMMT, in yeast (S7B Fig). Consistent with these bioinformatic results, previous research showed that YEM1L1, the human homolog of YME1, controls IMMT [10].

**Fig 7:**
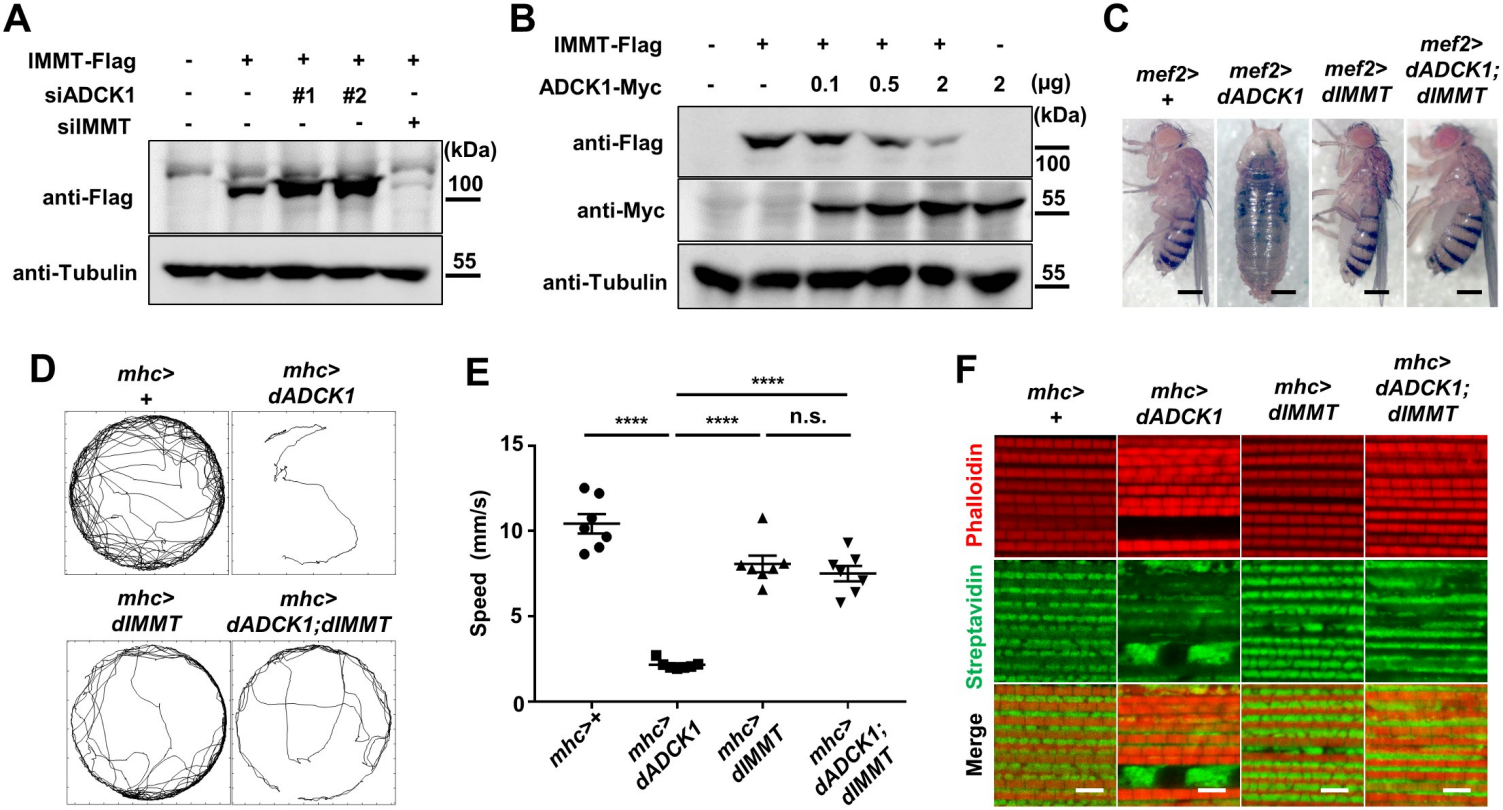
IMMT is a downstream target of ADCK1. (A) HEK293T cells were transfected with scramble siRNA (Scr), siADCK1s (#1 and #2), or siIMMT as indicated. WCL were prepared and analyzed for immunoblot with anti-Flag and anti-tubulin antibodies. (B) HEK293T cells were transfected with IMMT-Flag and ADCK1-Myc as indicated. WCL were prepared and analyzed for immunoblot analyses with anti-Flag, anti-Myc and anti-tubulin antibodies. (C) Whole body images of the flies with indicated genotypes. Scale bars, 0.5 mm. (D) Movement trajectories of the adult flies with indicated genotypes. (E) Comparison of the mean value of walking speed with indicated genotypes, n = 7. ****, p<0.0001; n.s., not significant by unpaired t-test. (F) Fluorescent confocal microscopy images of the adult thoraces with indicated genotypes. Streptavidin (green) and phalloidin (red) staining were used to mark mitochondria and muscle actin fibers, respectively. Scale bars, 5 μm.

In order to identify whether ADCK1 and IMMT genetically interact with each other, we expressed IMMT upon knockdown or over-expression of ADCK1 and determined their interactions using immunoblot analyses. When we over-expressed IMMT together with *ADCK1* knockdown, we observed increased protein levels of IMMT compared to the sole over-expression of IMMT (Fig 7A). Consistently, once IMMT and ADCK1 were over-expressed simultaneously, we observed that the protein level of IMMT was decreased depending on the level of ADCK1 (Fig 7B). From this observation, we confirmed the antagonistic relationship between ADCK1 and IMMT. Furthermore, we confirmed that the fly over-expressing both dADCK1 and dIMMT developed normally into the adult stage (Fig 7C). As the over-expression of dIMMT rescued the pupal lethality induced by dADCK1 over-expression, dIMMT could be a downstream target of dADCK1 just as dYME1L1. To further validate this finding, we analyzed the locomotor activity of the flies with dADCK1 over-expression, dIMMT over-expression or both using *mhc-Gal4* driver. The locomotive activity of the *dADCK1* over-expression flies was significantly decreased. However, the flies with simultaneous over-expression of dADCK1 and dIMMT displayed a similar outcome to that of the flies over-expressing dIMMT (Fig 7D and 7E). Finally, by observing the muscle structure and mitochondria morphology in the thorax of adult flies, we discovered that simultaneous over-expression of dADCK1 and dIMMT partially rescued the phenotypes of the *dADCK1* over-expressing flies (Fig 7F). Consummating these experimental data, we confirmed that IMMT functions downstream of ADCK1.

### OPA1 is another downstream target of ADCK1

**Fig 8:**
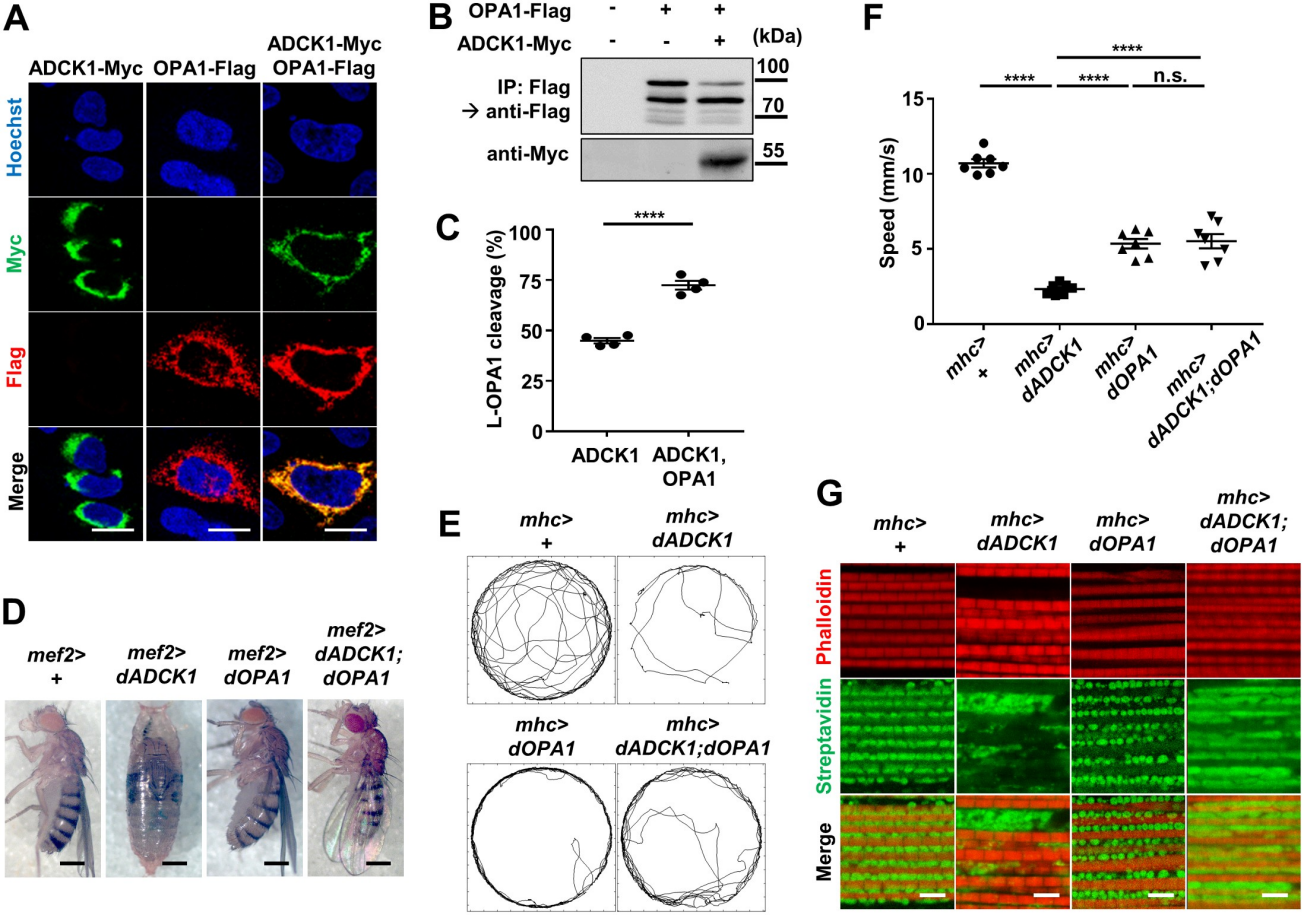
OPA1 is another downstream target of ADCK1. (A) Fluorescent confocal microscopy images of HeLa cells. HeLa cells were transfected with ADCK1-Myc and/or OPA1-Flag as indicated. The Myc-tagged ADCK1 proteins were immunolabeled with anti-Myc antibody (green) and OPA1 proteins were labeled with anti-Flag antibody (red). Hoechst (blue) was used for nuclei staining. Scale bars, 20 μm. (B) HEK293T cells were transfected with OPA1-Flag and/or ADCK1-Myc as indicated. WCL were used for detecting expression of ADCK1 using anti-Myc antibody. Immunoprecipitated samples were detected by anti-Flag antibody. (C) Quantification of the percentage of OPA1 protein cleavage, n = 4. ****, p<0.0001 by unpaired t-test. (D) Whole body images of the flies with indicated genotypes. Scale bars, 0.5 mm. (E) Movement trajectories of the adult flies with indicated genotypes. (F) Comparison of the means of walking speed for the flies with indicated genotypes, n = 7. ****, p<0.0001; n.s., not significant by unpaired t-test. (G) Fluorescent confocal microscopy images of the adult fly thoraces with indicated genotypes. Streptavidin (green) and phalloidin (red) staining were used to mark mitochondria and muscle actin fibers, respectively. Scale bars, 5 μm.

The increased mitochondrial fusion/fission due to the knockdown or the over-expression of ADCK1 was shown in previous experiments (Fig 5). Moreover, we confirmed that *dYME1L1* knockdown rescued the phenotypes of the *ADCK1* over-expression fly, such as developmental lethality, locomotive defects and mitochondrial abnormalities (Fig 6). Thus, we predicted that ADCK1 and YME1L1 would interact with other regulatory proteins to control mitochondrial fusion and fission. Through our effort to screen the interactions between ADCK1 and the proteins that are involved in mitochondrial fusion or fission in HeLa cells, we discovered that OPA1 rescues both the mitochondria clustering and increased fission phenotypes induced by ADCK1 over-expression (Fig 8A). Other proteins involved in mitochondrial fusion, MFN1 and MFN2, or proteins involved in mitochondrial fission, FIS1 and DNM1L (also known as DRP1), were also over-expressed with ADCK1. However, no significant changes were induced by them in the abnormal phenotypes of the ADCK1 over-expression (S8A and S8B Fig). Although we could confirm the genetic interaction of ADCK1 and the fusion protein OPA1, we could not identify a similar relationship between ADCK1 and fission proteins. For example, we studied whether the mitochondrial fission phenotypes of ADCK1 over-expression are dependent on DNM1L by expressing a dominant negative form of DNM1L [38]. As a result, the increased fission phenotypes due to ADCK1 over-expression were not affected by the expression of the DNM1L protein (S8B Fig).

Therefore, we concluded that OPA1 is involved in the phenotypes resulting from ADCK1 over-expression. OPA1 is a well-known protein involved in mitochondrial fusion by interacting with YME1L1 in which YME1L1 regulates the balance between long form of OPA1 (L-OPA1) and short form of OPA1 (S-OPA1) [39]. Thus, to identify whether ADCK1 affects the OPA1 protein processing by YME1L1, the ADCK1 and OPA1 were co-expressed and the cleavage pattern of OPA1 was analyzed through immunoblot analyses. The result demonstrated that the over-expression of both OPA1 and ADCK1 led to increased cleavage of L-OPA1 (Fig 8B and 8C). In addition, we observed that the transgenic flies over-expressing both dADCK1 and dOPA1 survived into adulthood (Fig 8D). Finally, simultaneous over-expression of dOPA1 partially rescued the locomotive defects (Fig 8E and 8F) and the mitochondrial anomalies in the muscle (Fig 8G) of the *dADCK1* over-expression flies. Collectively, these results supported that dOPA1 is another downstream target of dADCK1.

### Phenotypes induced by ADCK1 over-expression are independent of its kinase activity

The functions of ADCK1 discovered in our experiments were irrelevant to the kinase activity of the protein. Until now, ADCK1 was predicted to be a kinase, yet there were no available reports to confirm it. In our experiment, we engineered kinase-dead mutant forms of ADCK1 with substitutions of the key amino acids related to the phosphotransferase activity of ADCK1, such as A164G, K183I, D315A, and D338N mutations (S9A Fig) [1]. Over-expression of each mutant as well as the triple-mutation-containing form (K183I-D315A-D338N; 3KD) of ADCK1 still induced the same phenotypes similar to ADCK1 wild type, and thus we concluded that the phenotypes induced by ADCK1 are kinase-independent (S9B Fig). Moreover, no significant differences were detected between ADCK1 wild type and 3KD in the biochemical experiment testing for their inhibition on IMMT (S9C Fig). However, as the endogenous kinase might have affected the assay with the ADCK1 kinase-dead mutant, we performed an experiment of over-expressing the kinase-dead form of ADCK1 in *ADCK1* knocked down HeLa cells by expressing siRNA for *ADCK1* and were able to obtain the identical conclusion (S9D Fig). Based on these results, we again confirmed that ADCK1 regulates mitochondria in a kinase activity-independent manner.

## Discussion

In this study, we firstly identified the functions of ADCK1. By observing how over-expression of ADCK1 led to destruction of cristae structure and anomaly in mitochondrial functions, we discovered that ADCK1 maintains the cristae structure of the mitochondria by controlling IMMT. Moreover, we found that ADCK1 also involves in mitochondrial fusion/fission, since *ADCK1* knockdown increased fusion whereas its over-expression increased mitochondrial fission through OPA1. Furthermore, the regulation of IMMT and OPA1 by ADCK1 was critical in mitochondrial functions such as ATP production, ROS generation, and cell apoptosis. Secondly, we have discovered the ADCK1-dependent signaling pathway by using bioinformatics, fruit fly genetics and mammalian cell biology. Using PPI network analyses, comparative genomics and phylogenetic analysis, we predicted new interactions among specific mitochondrial proteins and ADCK1 and demonstrated their epistasis. As a result, we have revealed that ADCK1 controls IMMT and OPA1 through YME1L1. Thirdly, we observed the importance of ADCK1 in maintaining structures and functions of the muscular system in *Drosophila*. The flies with ADCK1 deficiency displayed developmental defects, abnormal wing structures, flight disabilities and decreased locomotive activities. Moreover, when we monitored changes in mitochondrial fusion/fission and cristae structure upon ADCK1 misexpression in the thorax muscles of flies, we found that the ADCK1 pathway is critical for the muscle-related abnormalities induced by mitochondrial dysfunctions.

The ADCK family has evolutionarily conserved AarF domains and is expected to function as serine/threonine or tyrosine kinase. Especially, the protein family was predicted to be related to ubiquinone biosynthesis. In addition, the ADCK family was predicted to be located at the inner or outer membrane of the mitochondria due to its transmembrane domain and mitochondrial targeting sequences. Until now, only ADCK3 and ADCK4 had been studied among the ADCK family and their involvement in ubiquinone biosynthesis has been validated. However, their role as kinases lacks evidence. In this study, we have initiated an investigation on the function of ADCK1, revealing its essential role in the regulation of mitochondrial functions and structures in fruit flies and mammal cells. Through phylogenetic and PPI network analysis, we differentiated ADCK1 and ADCK5 from ADCK3 and ADCK4, and further proved their difference by observing mitochondrial phenotypes in over-expression experiments performed in mammal cells. Separate from ADCK3 and ADCK4 that involve in ubiquinone biosynthesis, we found that the ADCK1 plays specific roles in mitochondrial fusion/fission and cristae maintenance. Although we have not thoroughly examined ADCK5, we predict that its function will be similar to that of ADCK1.

Through this study, we have confirmed that ADCK1 pathway is pivotal in controlling the functions and morphologies of mitochondria. To find the downstream target of ADCK1 among mitochondrial proteins listed from text mining, we performed RNAi-based genetic screenings to identify novel interactors that alter the over-expression phenotypes of ADCK1. In consequence, we found that YME1L1 genetically interacts with ADCK1. The fly genetics result proved that ADCK1 works in the direction of activating YME1L1 (Fig 6). As ADCK1 was originally predicted to be a kinase, we had expected that ADCK1 would affect the activity of YME1L1 by phosphorylation. However, we discovered that the phenotypes induced by ADCK1 were independent of its kinase activity (S9 Fig). Although we could not experimentally validate how ADCK1 regulates YME1L1, its mechanism can be predicted from earlier studies and our experimental results. YME1L1 and OMA1 are mitochondrial proteases that work antagonistically in cleaving OPA1 to control mitochondrial fusion/fission [30, 40]. ADCK1 may operate as an energy sensor that activates YME1L1 when ATP is abundant or activates OMA1 in shortage of ATP, consequently controlling mitochondrial structures and functions depending on ATP availability in the cell [24]. Overall, we propose the presence of the ADCK1-dependent signaling pathway composed of ADCK1-YME1L1- IMMT/OPA1 to control central functions of mitochondria, such as fusion/fission, cristae remodeling, production of ATP and ROS, and apoptosis (S10 Fig).

In addition, we propose that further research will be needed to find out whether ADCK1 pathway regulates mitochondrial quality control through mitophagy. We identified that fission occurred in the mitochondria between a normal and an abnormal section to recover normal mitochondria from the damaged ones with defective cristae structures upon *ADCK1* over-expression (Fig 6E). The separated abnormal section of mitochondria after mitochondrial fission will be eliminated through mitophagy [18]. Thus, the connection between ADCK1 and the mitophagy-controlling signaling molecules, such as ULK1, PINK1a nd Parkin that induce mitophagy [41–44], should be studied in the future.

In conclusion, our study revealed that ADCK1 plays critical roles in maintaining mitochondrial functions and structures. As malfunctioning of the ADCK1 pathway was confirmed to induce muscular dysfunctions, our research will be of help in finding the mechanism of pathogenesis and treatments for mitochondria-related muscular diseases.

## Materials and methods

### Fly stocks

All the fly stocks were maintained on a standard cornmeal medium at 25°C and 70% humidity with 12 hours/12 hours light/dark cycle. The fly medium composed of dextrose 1,260 g, yeast 900 g, cornmeal 635 g, agar 91 g, tegosept 132 ml, and propionic acid 84 ml in 18 L of food were manufactured by BIOMAX, Korea. *Cg-Gal4*, *Sgs-Gal4*, *tub-Gal4*, *mhc-Gal4*, *mef2-Gal4* and *nSyb-Gal4* driver strains were obtained from the Bloomington *Drosophila* Stock Center (BDSC), Indiana. Other flies were provided with generosity: *tub-Gal80*^*ts*^ was provided by Dr. Ron Davis (Scripps Research Institute, CA). *dADCK1 RNAi* (3608R-2) strain was obtained from National Institute of Genetics (NIG), Japan. *dADCK1 RNAi* (106695) and *dYME1L1 RNAi* (34282) strains were obtained from Vienna *Drosophila* Resource Center (VDRC), Austria. *dADCK1 RNAi* (42841), *dIMMT RNAi* (63994), *UAS-MitoGFP* (8442), *UAS-p35* (5072), *UAS-dSOD1* (24754), and *UAS-dSOD2* (24429) strains were obtained from BDSC. *UAS-dADCK1*, *UAS-dOPA1* and *UAS-dIMMT* were generated by microinjection of pUAST vector-cloned genes into *w*^*1118*^ embryos. Female flies were used in all experiments except for behavioral experiments in which male flies were used to eliminate the influences of fertilization and egg laying.

### Plasmids

The plasmids encoding human *ADCK1*, *ADCK3* and *ADCK4* were purchased from Addgene. The plasmids encoding human *ADCK2*, *ADCK5* and *IMMT* were purchased from 21C Frontier Human Gene Bank (KRIBB, Korea). The plasmids encoding *dADCK1*, *dADCK3/4* and *dADCK5* were purchased from *Drosophila* Genomics Resource Center (DGRC, IN). The cDNAs encoding *ADCK1*, *ADCK2*, *ADCK3*, *ADCK4*, *ADCK5*, *dADCK1*, *dADCK3/4*, and *dADCK5* were cloned into pcDNA3.1/Myc-His vector (Invitrogen, MA). The cDNA encoding *IMMT* was cloned into p3×FLAG-CMV14 vector (Sigma, OH). The plasmids encoding DRP1-6Myc, MFN1-10Myc, and MFN2-16Myc were generous gifts from Dr. David C. Chan (California Institute of Technology, CA). The plasmid encoding OPA1-Flag was a generous gift from Dr. Hayoung Lee (Chungnam National University, Korea).

### Antibodies

Mouse anti-Myc (Medical and Biological Laboratories, cat#M192-3, Japan), rabbit anti-Flag (Cell Signaling Technology, cat#2368, MA), mouse anti-tubulin (DSHB, cat#E7, IA), rabbit anti-COXIV (Cell Signaling Technology, cat#4850, MA), rabbit anti-ADCK1 (Thermo Fisher Scientific, cat# PA5-22170, MA) and MTC02 (Abcam, UK) were used for immunocytochemistry or immunoblotting.

### Generation of *dADCK1* mutant flies

The guide RNA1 and guide RNA2 sequences were identified using flyCRISPR Optimal Target Finder [45]. Target sequences were as the following. gRNA1: ‘5-GGTACGATTATTCCAATCTCTGG-3’. gRNA2: ‘5-GGATCCGGAATCAGACTGCTTGG-3’. *dADCK1*cDNA was cloned into the BbsI site of pU6-BbsIchiRNA(DGRC) and the plasmid was purified with a midi prep kit (Qiagen, Netherlands). Injection mixes were prepared with 500 ng/μl of phsp70-Cas9 and 250 ng/μl of gRNA plasmids, and the constructs were injected into *w*^*1118*^ embryos.

### Flight ability assay

To check the flight ability, 30-day-old flies of 30 per group were collected and dropped in mass cylinder of 420 mm in height, covered with glycerol on the inner surface. Next, the numbers of flies that flew and landed on inner walls before hitting the bottom were counted separately from the flies that could not fly and dropped to bottom. The flies with *tub-Gal80*^*ts*^*/tub-Gal4* driver were raised at 18°C, and from 2 to 3 days after birth they were shifted to 30°C or 18°C for seven days and finally placed at 25°C to measure the flight ability.

### Video-tracked behavioral analysis

A camera was installed inside a 675 mm × 440 mm × 410 mm sized insulated box and an adult fly 5 to 6 days after birth were acclimated at 25°C for 1 hour. Next, its free movement inside a transparent and circular arena with 2 mm in height and 600 mm in diameter was recorded with 30.06 fps (frame per second). The recorded video was converted to MATLAB file using Ctrax and the behavioral microarray toolbox was utilized to obtain the mean speed and trajectory graph. The flies with *tub-Gal80*^*ts*^*/tub-Gal4* driver were raised at 18°C, and from 2 to 3 days after birth they were shifted to 30°C or 18°C for seven days and finally placed at 25°C to measure the assay.

### Life span assay

The life span assay was prepared by obtaining 100 eggs per group over 1 hour, from each bottle of grape juice medium with 100 virgin female flies and 20 male flies. For the mutants, *Cyo-GFP* balancer was used to collect fly embryos with homozygote mutant alleles. The numbers of first/second/third instar larvae and pupae developed from the eggs were measured at specific time point of after egg laying (AEL). The life span assay in the adult fly was prepared by raising 4 sets of newborn F2 progenies with equal ratio of both sexes per groups, at 30°C on regular medium. The flies were transferred to new medium every day and the number of dead flies was counted.

### Immunohistochemistry on fly thorax, salivary gland tissues

To observe the adult fly thorax, fly head was removed from the adult fly 20- to 21-day-old and the remaining body was fixed in 4% paraformaldehyde (PFA) for 1 hour and washed 3 times in 0.1% PBST (PBS + Triton X-100) for 15 minutes. Then it was dissected in 1× PBS to obtain thorax. The separated thorax was permeabilized with 0.5% PBST for 5 minutes. The permeabilized tissue was washed 3 times with 0.1% PBST for 10 minutes and incubated with 3% bovine serum albumin and 10% normal goat serum in 0.1% PBST for 30 minutes at room temperature (RT). Alexa Fluor 488-conjugated streptavidin (Sigma, S11223, 1:150) and phalloidin-tetramethylrhodamine B isothiocyanate (Sigma, P1951, 1:500) were applied for overnight at 4°C to stain mitochondria and thorax muscle actin fibers, respectively. On the next day, the tissues were washed 3 times in 0.1% PBST for 15 minutes. Finally, the samples were washed twice in PBS for 10 minutes each and mounted on a slide glass with SlowFade mounting solution (Invitrogen, CA, ID: S36936). The slides were observed with LSM710 laser scanning confocal microscopy (Carl Zeiss, Germany) under 1,000 × magnification. All washing procedure was performed using nutator.

To examine the mitochondria in the salivary gland, late third instar larvae were fixed in 4% PFA for 20 minutes and washed twice with 0.1% PBST for 15 minutes each. Then the larvae were dissected in 1×PBS to obtain the salivary gland. The tissue was permeabilized and stained with phalloidin-tetramethylrhodamine B isothiocyanate (1:500) and Hoechst (Sigma, 33258, 1:500) for 20 minutes at RT. Next, the samples were washed 3 times in PBS for 15 minutes each and mounted on a slide glass with SlowFade mounting solution.

### Measurement of mitochondrial membrane potential

To measure mitochondrial membrane potentials, a 20- to 21-day-old adult fly was dissected in Schneider’s *Drosophila* medium and stained with 2.5 nM tetramethylrhodamine methyl ester (TMRM, Molecular Probes, MA) for 20 minutes. Then, it was washed 3 times with PBS for 10 minutes each and fixed in 4% PFA for 40 minutes. Finally, the samples were washed twice in PBS for 10 minutes each and mounted on a slide glass with SlowFade mounting solu. The slides were observed with LSM710 laser scanning confocal microscopy (Carl Zeiss, Germany) under 1,500× magnification.

### ATP assay

The thoraces of ten 20- to 21-day-old flies were homogenized in cell lysis reagent in ATP Bioluminescence Assay Kit HS II (Roche, cat. no. 11 699 709 001, Swiss). The luminescence was measured by Infinite M200 Pro (Tecan, Swiss), and the results were compared to standards. The relative ATP level was then calculated by dividing the luminescence by the total protein concentration, which was determined by Bradford assay.

### DHE staining

To observe the ROS level, flies were raised in 18°C, and from 2 to 3 days after birth they were moved to 30°C or 18°C for seven days and finally placed at 25°C to be dissected in PBS. After incubation for 7 minutes with 30 μM DHE in a dark chamber, the samples were washed twice in PBS, fixed in 4% PFA for 8 minutes, and washed 3 times with PBS for 15 minutes each.

### TUNEL assay

To monitor apoptosis, flies were placed at 25°C to cut out the heads and were fixed in 4% PFA for 1 hour. The fixed thorax was washed 3 times with 0.1% PBST for 15 minutes each. 0.1 M sodium citrate in 0.1% PBST was applied before incubating the sample at 65°C for 30 minutes. The samples were washed 3 times with PBS for 15 minutes each. For the TUNEL reaction, the samples were incubated in the mixture of 2 μl of TUNEL enzyme (TMR red, 12156792910, Roche, Swiss) and 27 μl of buffer at 38°C for 2 hours. After the reaction, the samples were washed twice with 0.1% PBST for 15 minutes each, and Hoechst was applied before incubating the samples for 15 minutes at RT. Next, the solution was removed and the samples were washed 3 times with 0.1% PBST for 15 minutes each.

### Quantitative RT-PCR

Total RNAs from the thoraces of 2- to 3-day-old flies were extracted using Trizol Reagent (Invitrogen) and reversely transcribed by M-MLV Reverse Transcriptase (Promega). To check the inhibition of dADCK1 expression in dADCK1 RNAi lines, 7 thoraces were used. Quantitative real-time PCR was performed using SYBR Premix Ex Taq (Takara) on Prism 7000 Real-Time PCR System (ABI). PCR with primer sets of the following sequences: 5’-CAGGGCCTGACCAAAGTCAA-3’ for dADCK1-F, 5’-CATAACCCAGAAGGCGGTCA-3’ for dADCK1-R, 5’-GCGCTTCTTGGAGGAGACGCCG-3’for RP49-F and 5’-GCTTCAACATGACCATCCGCCC-3’ for RP49-R.

### Cell culture and transfection

HeLa and HEK293T cells were grown in Dulbecco’s-modified Eagle’s medium supplemented with 10% fetal bovine serum (Thermo Fisher Scientific, MA) at 37°C in a humidified atmosphere with 5% CO_2_. Transfection of mammalian expression plasmids was performed using polyethylenimine (Sigma, OH) or Lipofectamine Plus Reagent (Invitrogen, MA) according to the manufacturer’s instruction. siRNAs (siScramble and siADCK1-#1, #2, and siIMMT) were purchased from Bioneer (Korea). siRNA was transfected using Lipofectamine RNAiMAX transfection reagent (Invitrogen, MA) according to the manufacturer’s instruction. Co-transfection of siRNA and plasmids was performed using Lipofectamine 2000 reagent (Invitrogen, MA) according to the manufacturer’s instruction.

### Preparation of cell lysates, immunoprecipitation and immunoblotting

For preparation of cell lysates, cells were washed with cold PBS and lysed with Buffer A (20 mM Tris, pH 7.5, 100 mM NaCl, 1 mM EDTA, 2 mM EGTA, 50 mM β-glycerophosphate, 50 mM NaF, 2 mM DTT, 1 mM PMSF, 5 μg/ml leupeptin, 1 μg/ml pepstatin A and 1% Triton X-100). After cell lysis, cell lysates were centrifuged at 16,100×g for 30 minutes. The supernatant was subjected to immunoprecipitation and immunoblotting according to standard procedures. Anti-FLAG M2 Affinity Gel (Sigma, cat#A2220) was used for immunoprecipitation of Flag-fused proteins. Total protein levels were quantified using Bradford assay according to the manufacturer’s instruction. The immunoblots were developed and visualized using LAS-4000 (Fujifilm, Japan).

### Immunofluorescence analysis for cultured cells

HeLa cells were subcultured on coverslips in a 12-well tissue culture plate. Cells were washed once with PBS, fixed in 4% PFA for 15 minutes, and permeabilized with 0.5% PBST for 10 minutes. Then, the cells were washed with 0.1% PBST and incubated in blocking solution (3% BSA and 1% normal goat serum in PBST) for 1 hour. Primary antibodies were added to blocking solution (1:200) and the cells were incubated overnight at 4°C. After washing with PBST six times, cells were incubated with appropriate secondary antibodies (1:200) and Hoechst (33342, 1:2,000) in blocking solution for 1 hour at RT. The antibody-labeled cells were washed with PBS-T for six times and were mounted with mounting solution [100 mg/ml 1,4-diazabicyclo[2.2.2] octane (DABCO) in 90% glycerol]. The slides were observed with LSM710 laser scanning confocal microscopy (Carl Zeiss, Germany).

### Protein-protein interaction network analysis

The protein-protein interaction (PPI) networks for the ADCK family proteins were constructed. The interacting proteins were extracted from interactome database, BioGRID and IntAct. The Cytoscape 3 was used to construct, visualize and analyze the networks. Based on the obtained data from each interactome database, we developed PPI networks. By integrating all the networks generated, the entire human protein-scale network was constructed. Next, the neighborhood nodes that interact with ADCK1 were extracted into a small network. Each member of the ADCK families underwent similar interaction network constructions. Finally, all ADCK family protein networks were combined to form the ADCK family protein interaction network. The yeast homolog PPI networks of ADCK1 and IMMT were developed in an identical manner. The orthologues of the ADCK1 and IMMT were extracted from HomoloGene in the NCBI and Pan-taxonomic compara in the Ensembl.

### Phylogenetic analysis and comparative genomics

The orthologues of the ADCK family proteins were extracted from HomoloGene in the NCBI and Pan-taxonomic compara in the Ensembl. We aligned known ADCK1 paralogue sequences from human to fruit fly and analyzed the sequences. The protein sequences of the ADCK1 orthologues for human (*Homo sapiens*), cow (*Bos Taurus*), mouse (*Mus musculus*), chicken (*Gallus gallus*), zebra fish (*Danio rerio*), and fruit fly (*Drosophila melanogaster*) were downloaded from Uniprot. The Clustal Omega was used to generate multiple sequence alignments. Aligned sequences were visualized and annotated with Jalview.

### Text mining to find candidates

The human protein annotation information was obtained from UniProt, and the gene ontology information was obtained from the Gene Ontology (GO) database. The information for human and fruit fly homologs was obtained from HomoloGene in the NCBI and Pan-taxonomic compara in the Ensembl. Searching and matching for text mining were performed using Python program and Linux shell script.

### Transmission electron microscopy (TEM)

HeLa cells and fly thoraces were fixed with 3% glutaraldehyde in 0.1 M cacodylate buffer (pH 7.2)-containing 0.1% CaCl_2_ for 3 hours at RT. They were washed five times with 0.1 M cacodylate buffer at 4°C. Then, they were postfixed with 1% OsO_4_ in 0.1 M cacodylate buffer-containing 0.1% CaCl_2_ for 2 hours at 4°C. After rinsing with cold distilled water, the cells were dehydrated slowly with an ethanol series and propylene oxide at 4°C. The samples were embedded in Embed-812 (EMS, PA). After polymerization of the resin at 60°C for 36 hours, serial sections were performed with a diamond knife on an ULTRACUT UC7 ultramicrotome (Leica, Germany) and mounted on formvar-coated slot grids. Sections were stained with 4% uranyl acetate for 10 minutes and lead citrate for 7 minutes. They were observed using a Tecnai G2 Spirit Twin transmission electron microscope (FEI, OR).

## Supporting information

S1 Fig ADCK1 was identified as a novel mitochondrial regulatory protein. (A) A flow chart of the text mining processes to find a novel mitochondrial regulatory protein. (B) Sequence alignments of the *ADCK1* genes in several organisms (human, cow, mouse, chicken, zebra fish, and fruit fly). The part shown in a red dotted box indicates 349–473 amino acid positions deleted in *dADCK1*^*del*^.
(TIF)

S2 Fig The knockdown efficiencies of various *dADCK1* RNAi lines. (A) Whole body images of the flies with indicated genotypes. *dADCK1* knockdown using *tub-Gal4* driver for ubiquitous expression. Scale bars, 0.5 mm. (B) Comparison of *dADCK1* mRNA expression levels in the flies with indicated genotypes, n = 5. ****, p<0.0001 by unpaired t-test. (C) Movement trajectories of the adult flies with indicated genotypes. (D) Comparison of the means of walking speed for the flies with indicated genotypes, n = 7. ****, p<0.0001 by unpaired t-test.
(TIF)

S3 Fig *dADCK1* knockdown in fat body and neuronal tissues. (A) Comparison of the flight ability for the flies with indicated genotypes. n = 20. ****, n.s., not significant by unpaired t-test. (B) Movement trajectories of the adult flies with indicated genotypes (C) Comparison of the means of walking speed for the flies with indicated genotypes, n = 7. n.s., not significant by unpaired t-test.
(TIF)

S4 Fig *ADCK1* knockdown or over-expression changes mitochondrial morphologies. (A) Fluorescent confocal microscopy images of HeLa cells. HeLa cells were transfected with siScr or siADCK1 as indicated. Mitochondria were labeled with anti-MTC02 antibody (green) and Hoechst (blue) was used for nuclei staining. Scale bars, 10 μm. (B) Comparison of the mitochondrial circularity between control HeLa cells and HeLa cells transfected with ADCK1. n = 7. ****, p<0.0001 by unpaired t-test. (C) Comparison of the distance between nearby mitochondria in controls and HeLa cells transfected with ADCK1. n = 10. ****, p<0.0001 by unpaired t-test.
(TIF)

S5 Fig A screening experiment to find genes that alter the over-expression phenotypes of dADCK1. A flow chart of the text mining processes to find novel genes that alters the over-expression phenotypes of dADCK1using text mining.
(TIF)

S6 Fig ADCK1 and ADCK5 over-expression induce abnormal mitochondrial morphologies. (A) The phylogenetic tree of the ADCK family proteins of human and *Drosophila*. ADCK1 and ADCK5 showed evolutionary proximity in both human and *Drosophila*. The numbers represent the number of differences between sequences. (B-C) Fluorescent confocal images of the ADCK family proteins of human and *Drosophila* expressed in HeLa cells. HeLa cells were transfected with *ADCK* genes as indicated. The Myc-tagged ADCK family proteins were immunolabeled with anti-Myc antibody (green) and mitochondria were labeled with anti-MTC02 antibody (red). Hoechst (blue) was used for nuclei staining. Scale bars, 20 μm.
(TIF)

S7 Fig PPI network analyses for ADCK1. (A) A simplified human PPI network of ADCK family proteins with interacting proteins. (B) A simplified yeast PPI network of MCP2, the homolog of ADCK1, and MIC60, the homolog of IMMT.
(TIF)

S8 Fig Simultaneous expression of ADCK1 and mitochondrial fusion/fission proteins except OPA1 shows no significant changes in the phenotypes induced by ADCK1. (A-B) Fluorescent confocal microscopy images of HeLa cells. HeLa cells were transfected with ADCK1 and MFN1-Myc, MFN2-Myc, FIS1-Flag, DNM1L-Myc wild type, or DNM1L-Myc K38A as indicated. The ADCK1 proteins were immunolabeled with anti-ADCK1 antibody (green) and MFN1, MFN2, FIS1, DNM1L proteins were labeled with anti-Myc or anti-Flag antibody (red). Hoechst (blue) was used for nuclear staining. Scale bars, 20 μm.
(TIF)

S9 Fig Phenotypes induced by ADCK1 over-expression are independent on its kinase activity. (A) A pairwise alignment of the kinase-related domains of ADCK1 and ADCK3. The arrows indicate the substituted positions to generate an ADCK1 kinase-dead form. (B) Fluorescent confocal images of the *ADCK1* mutants in HeLa cells. HeLa cells were transfected with *ADCK1* mutant constructs as indicated. The 3KD mutant contains triple mutations of K183I, D315A, and D338N. The Myc-tagged ADCK1 proteins were immunolabeled with anti-Myc antibody (red) and the mitochondria were labeled with anti-MTC02 antibody (green). Hoechst (blue) was used for nuclear staining. Scale bars, 20 μm. (C) HEK293T cells were transfected with IMMT-Flag and ADCK1-Myc (WT or 3KD) as indicated. WCL were prepared and analyzed for immunoblot with anti-Flag, anti-Myc and anti-tubulin antibodies. (D) Fluorescent confocal microscopy images of HeLa cells. HeLa cells were transfected with siADCK1 and ADCK1-Myc WT or ADCK1-Myc 3KD as indicated. The Myc-tagged ADCK1 proteins were immunolabeled with anti-Myc antibody (green) and the mitochondria were labeled with anti-COX IV antibody (red). Hoechst (blue) was used for nuclear staining. Scale bars, 20 μm.
(TIF)

S10 Fig A schematic model of ADCK1 pathway in mitochondria. Dysfunctions in ADCK1 pathway induce mitochondrial cristae defects, mitochondrial fusion/fission imbalance, increased ROS, and apoptosis.
(TIF)

S1 File Numerical supporting information. The numerical data for the graphs in the figures.
(XLSX)
