# Supplementary figures and images for: Drosophila ADCK1 is critical for maintaining mitochondrial structures and functions in the muscle
Source: PLoS Genet. 2019 May 24;15(5):e1008184. doi: 10.1371/journal.pgen.1008184 (PMC6553794; doi:10.1371/journal.pgen.1008184)

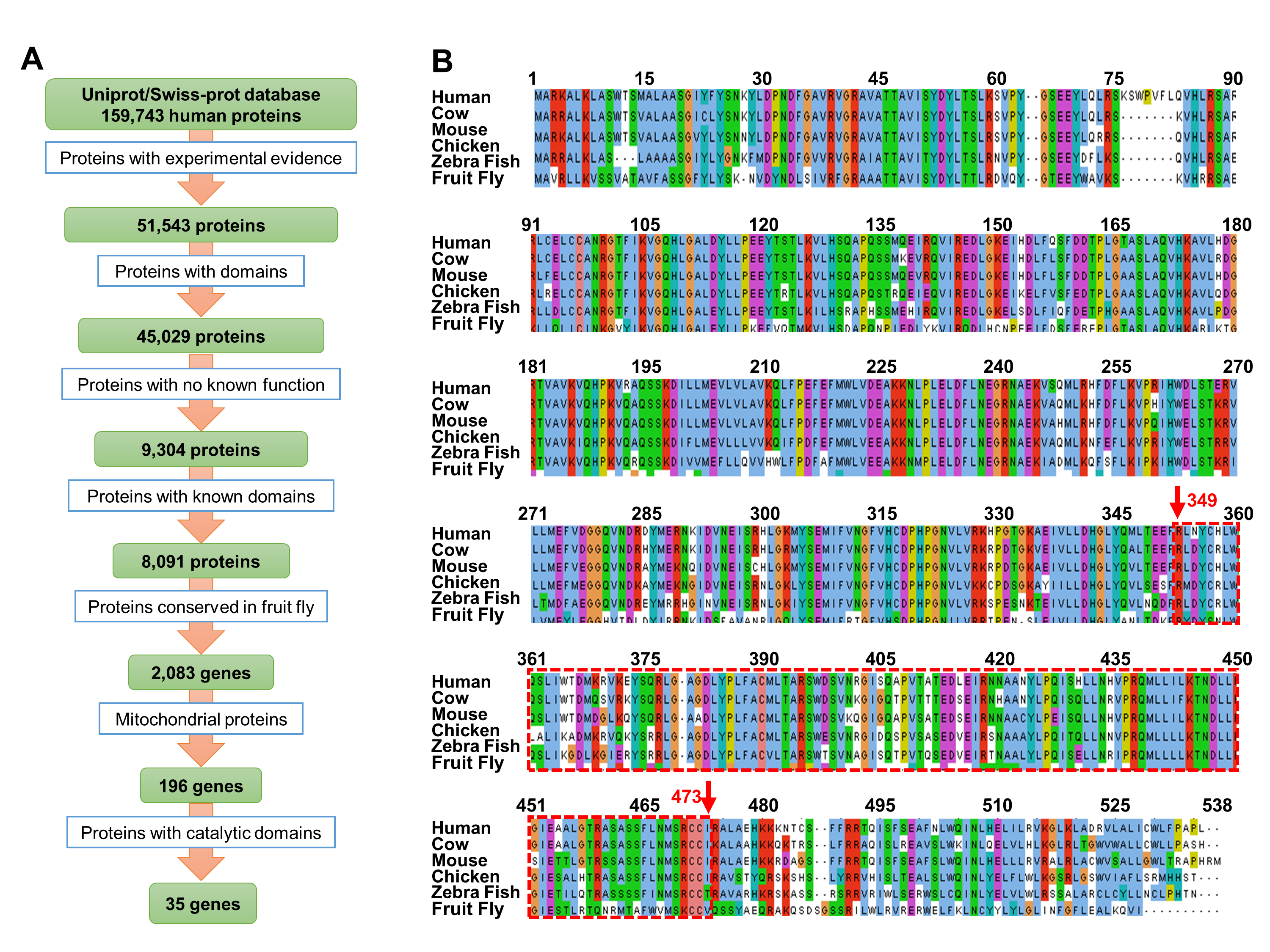

Supplement: S1 Fig — (A) A flow chart of the text mining processes to find a novel mitochondrial regulatory protein. (B) Sequence alignments of the ADCK1 genes in several organisms (human, cow, mouse, chicken, zebra fish, and fruit fly). The part shown in a red dotted box indicates 349–473 amino acid positions deleted in dADCK1del. (TIF) [file pgen.1008184.s001.tif]

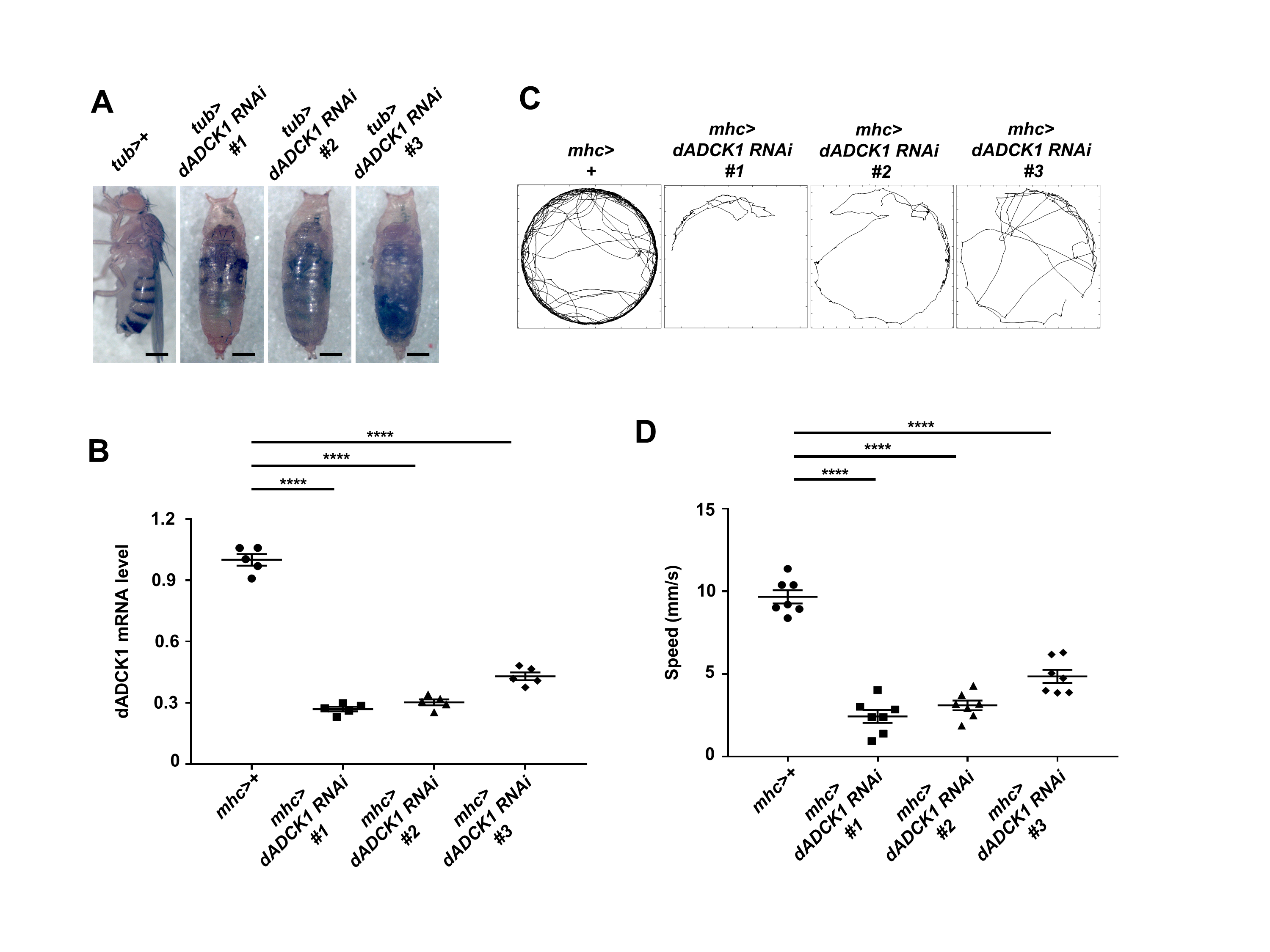

Supplement: S2 Fig — (A) Whole body images of the flies with indicated genotypes. dADCK1 knockdown using tub-Gal4 driver for ubiquitous expression. Scale bars, 0.5 mm. (B) Comparison of dADCK1 mRNA expression levels in the flies with indicated genotypes, n = 5. ****, p<0.0001 by unpaired t-test. (C) Movement trajectories of the adult flies with indicated genotypes. (D) Comparison of the means of walking speed for the flies with indicated genotypes, n = 7. ****, p<0.0001 by unpaired t-test. (TIF) [file pgen.1008184.s002.tif]

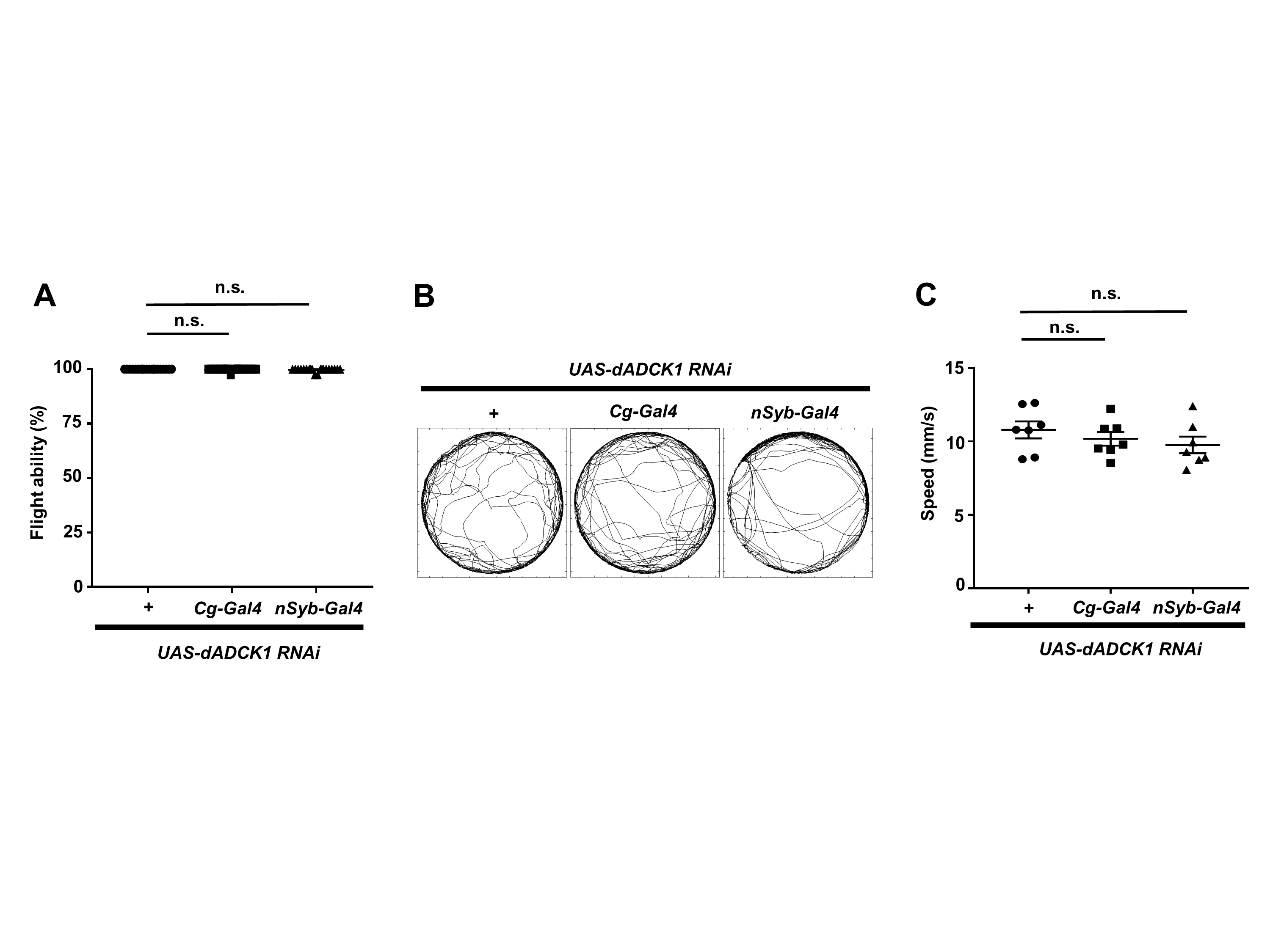

Supplement: S3 Fig — (A) Comparison of the flight ability for the flies with indicated genotypes. n = 20. ****, n.s., not significant by unpaired t-test. (B) Movement trajectories of the adult flies with indicated genotypes (C) Comparison of the means of walking speed for the flies with indicated genotypes, n = 7. n.s., not significant by unpaired t-test. (TIF) [file pgen.1008184.s003.tif]

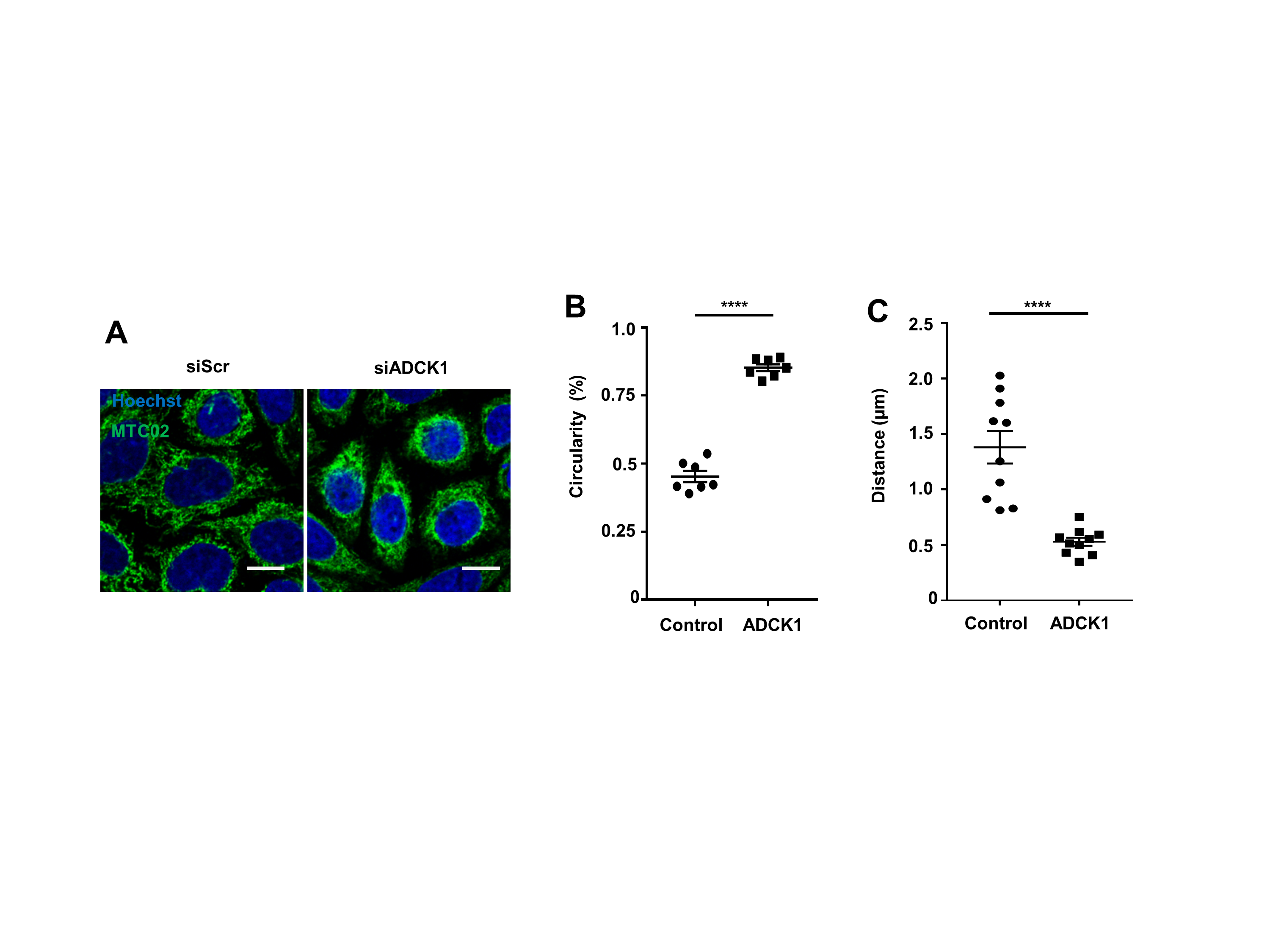

Supplement: S4 Fig — (A) Fluorescent confocal microscopy images of HeLa cells. HeLa cells were transfected with siScr or siADCK1 as indicated. Mitochondria were labeled with anti-MTC02 antibody (green) and Hoechst (blue) was used for nuclei staining. Scale bars, 10 μm. (B) Comparison of the mitochondrial circularity between control HeLa cells and HeLa cells transfected with ADCK1. n = 7. ****, p<0.0001 by unpaired t-test. (C) Comparison of the distance between nearby mitochondria in controls and HeLa cells transfected with ADCK1. n = 10. ****, p<0.0001 by unpaired t-test. (TIF) [file pgen.1008184.s004.tif]

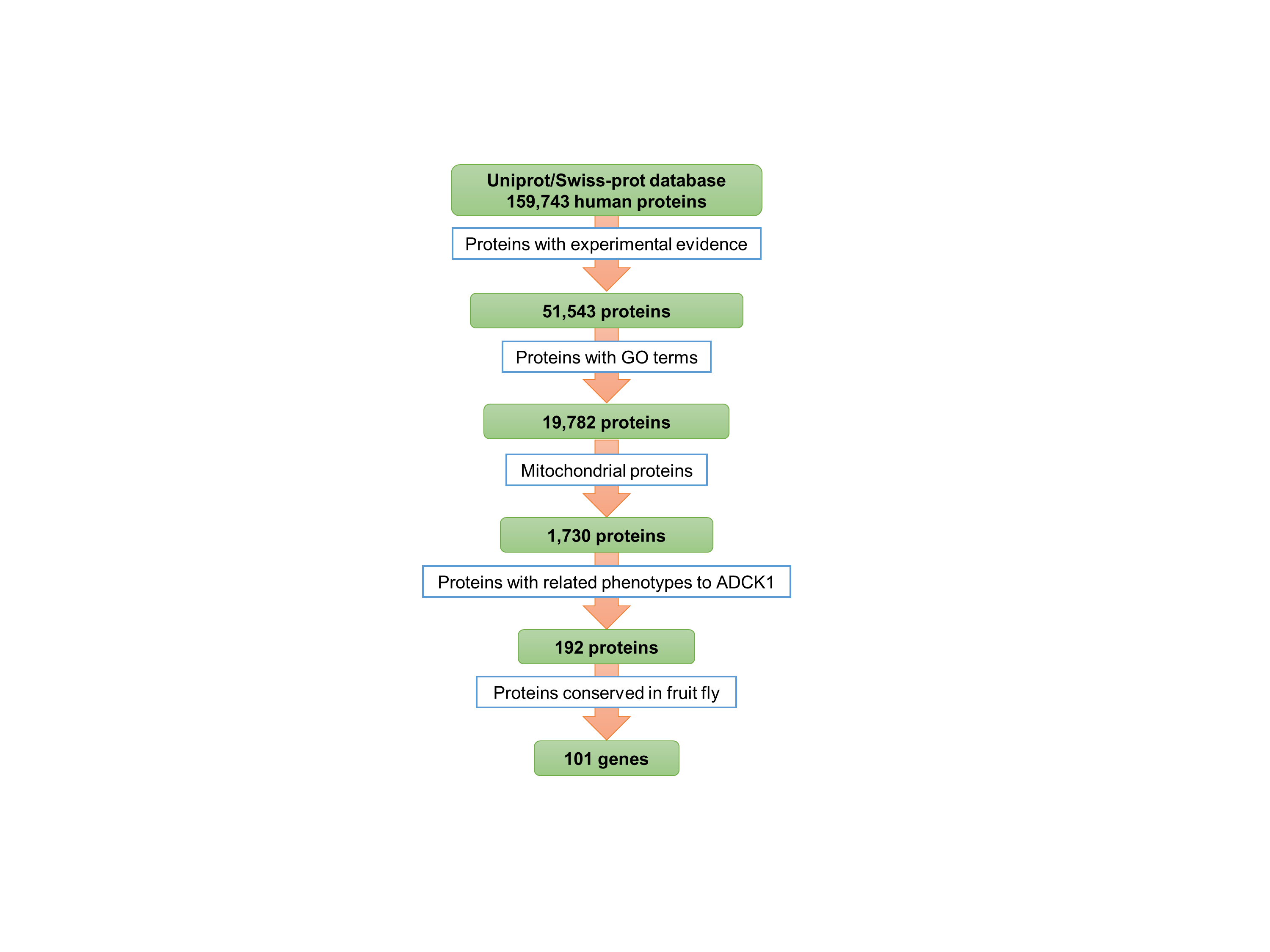

Supplement: S5 Fig — A flow chart of the text mining processes to find novel genes that alters the over-expression phenotypes of dADCK1using text mining. (TIF) [file pgen.1008184.s005.tif]

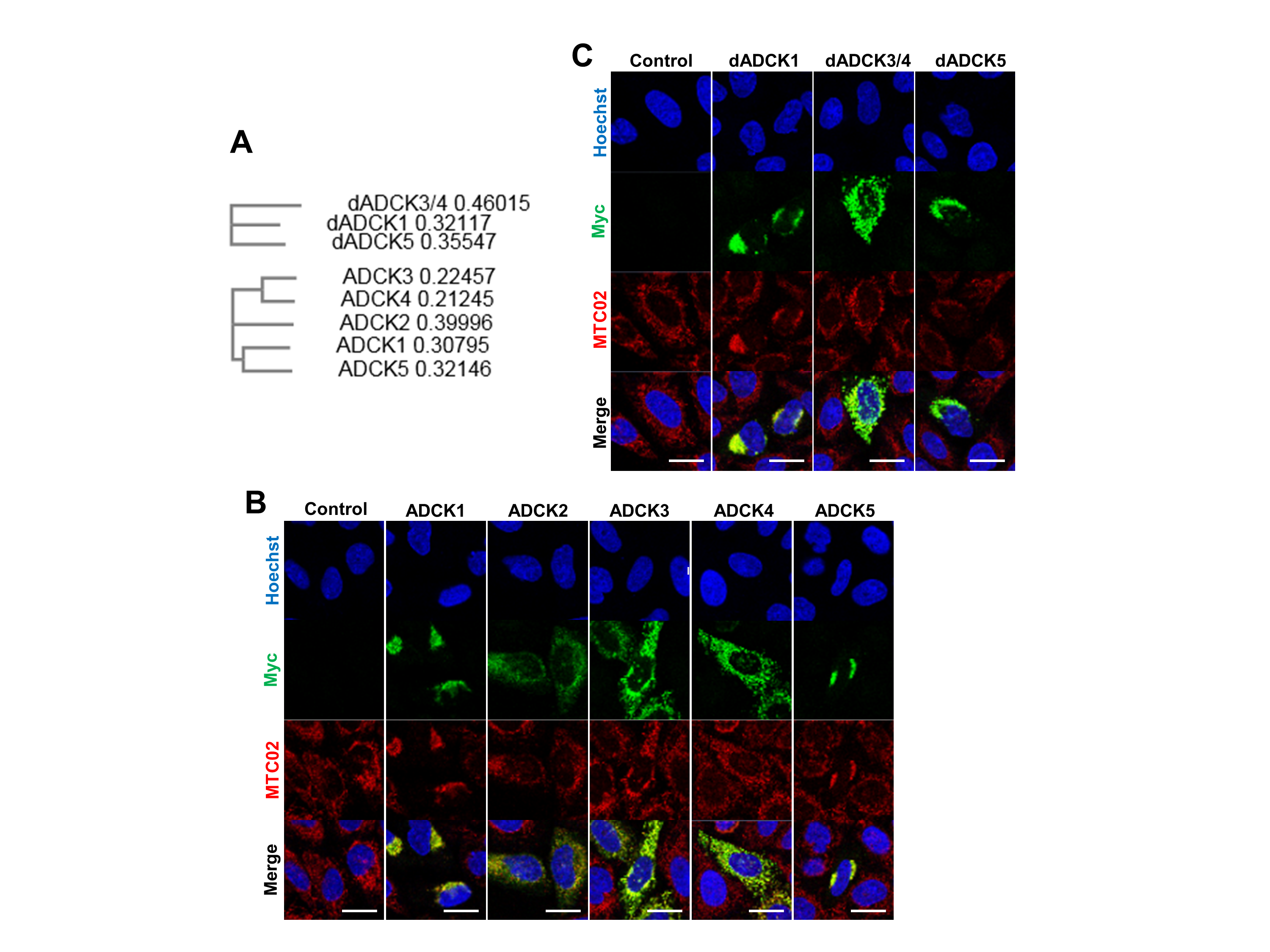

Supplement: S6 Fig — (A) The phylogenetic tree of the ADCK family proteins of human and Drosophila. ADCK1 and ADCK5 showed evolutionary proximity in both human and Drosophila. The numbers represent the number of differences between sequences. (B-C) Fluorescent confocal images of the ADCK family proteins of human and Drosophila expressed in HeLa cells. HeLa cells were transfected with ADCK genes as indicated. The Myc-tagged ADCK family proteins were immunolabeled with anti-Myc antibody (green) and mitochondria were labeled with anti-MTC02 antibody (red). Hoechst (blue) was used for nuclei staining. Scale bars, 20 μm. (TIF) [file pgen.1008184.s006.tif]

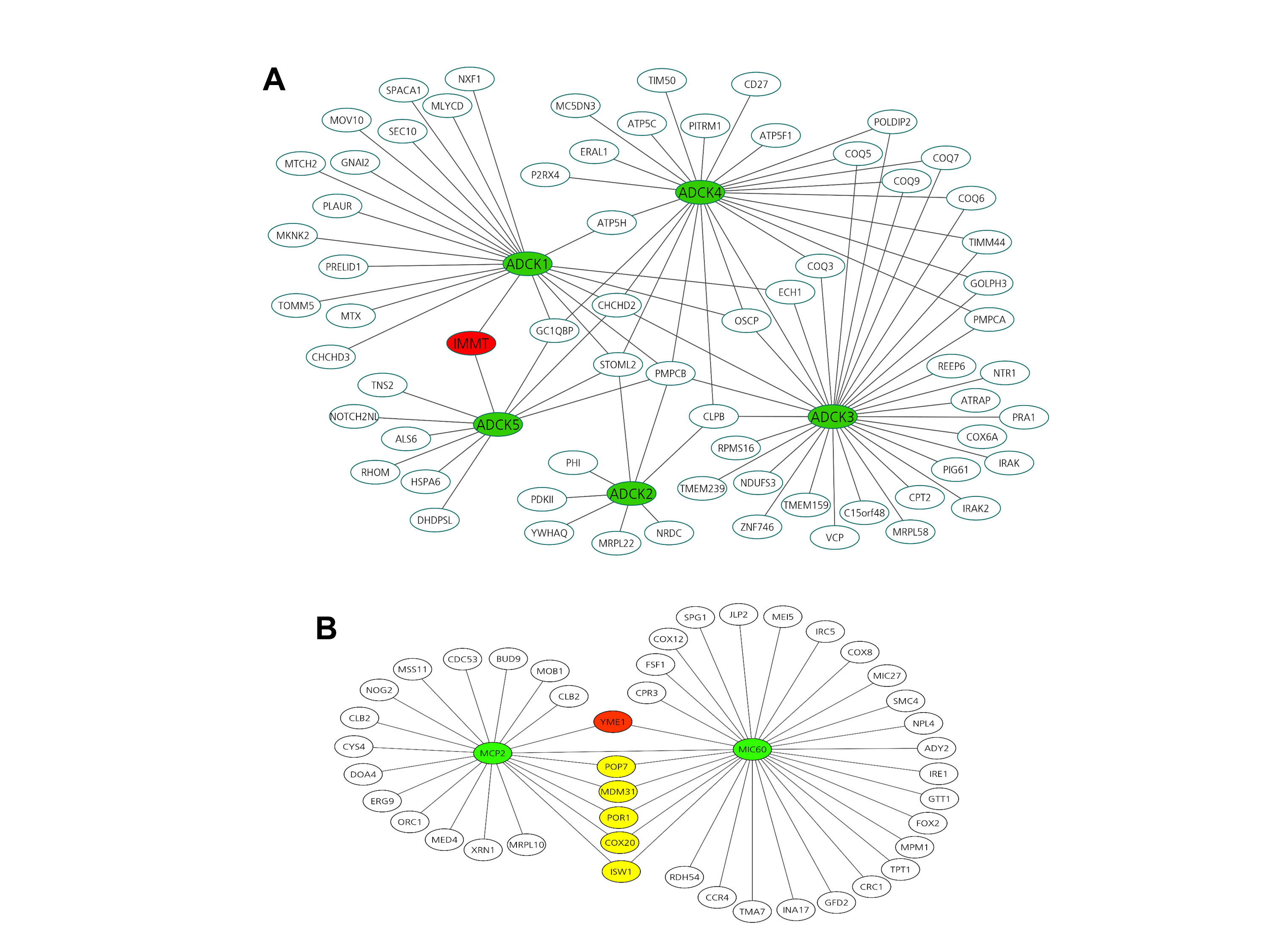

Supplement: S7 Fig — (A) A simplified human PPI network of ADCK family proteins with interacting proteins. (B) A simplified yeast PPI network of MCP2, the homolog of ADCK1, and MIC60, the homolog of IMMT. (TIF) [file pgen.1008184.s007.tif]

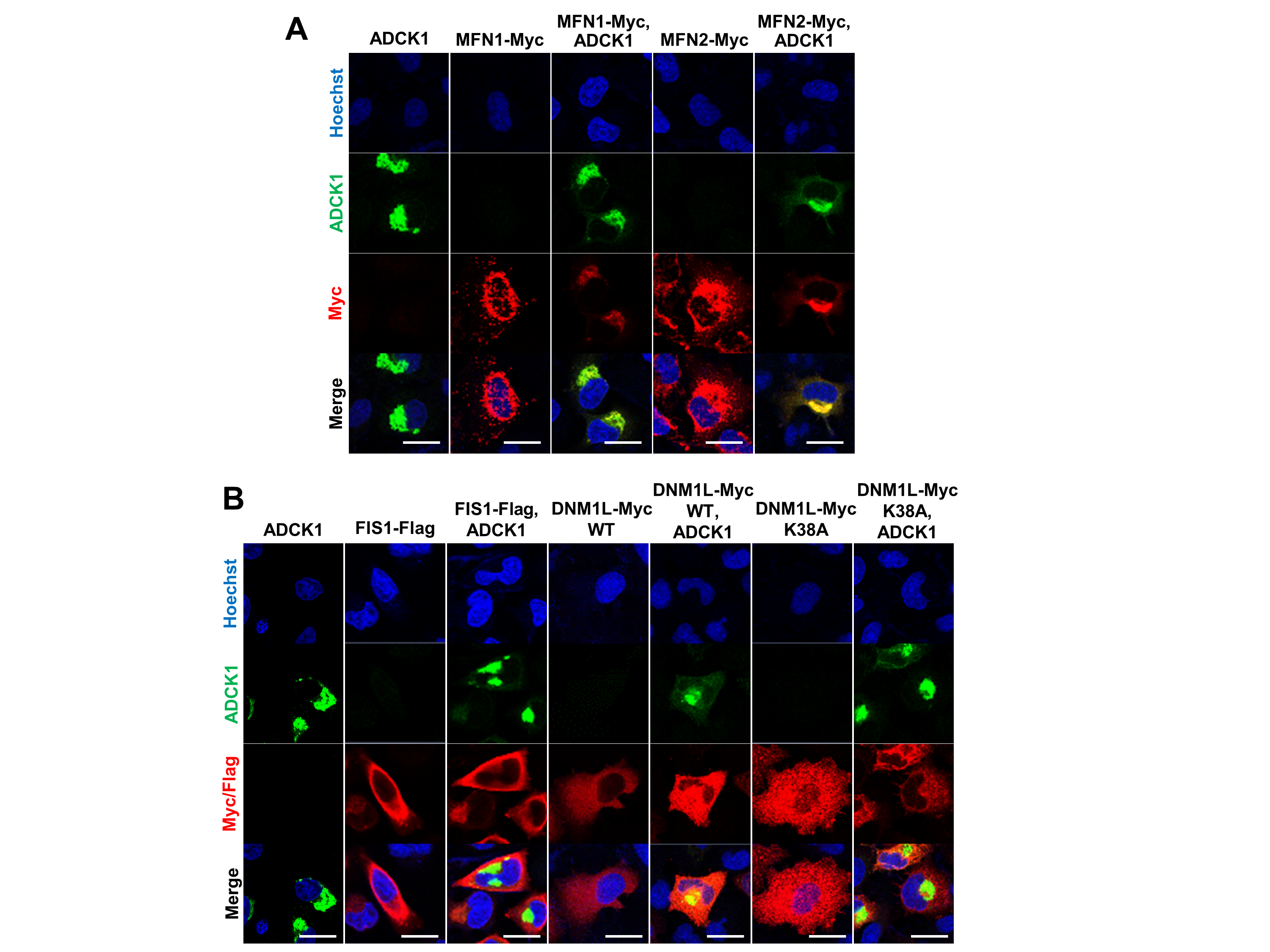

Supplement: S8 Fig — (A-B) Fluorescent confocal microscopy images of HeLa cells. HeLa cells were transfected with ADCK1 and MFN1-Myc, MFN2-Myc, FIS1-Flag, DNM1L-Myc wild type, or DNM1L-Myc K38A as indicated. The ADCK1 proteins were immunolabeled with anti-ADCK1 antibody (green) and MFN1, MFN2, FIS1, DNM1L proteins were labeled with anti-Myc or anti-Flag antibody (red). Hoechst (blue) was used for nuclear staining. Scale bars, 20 μm. (TIF) [file pgen.1008184.s008.tif]

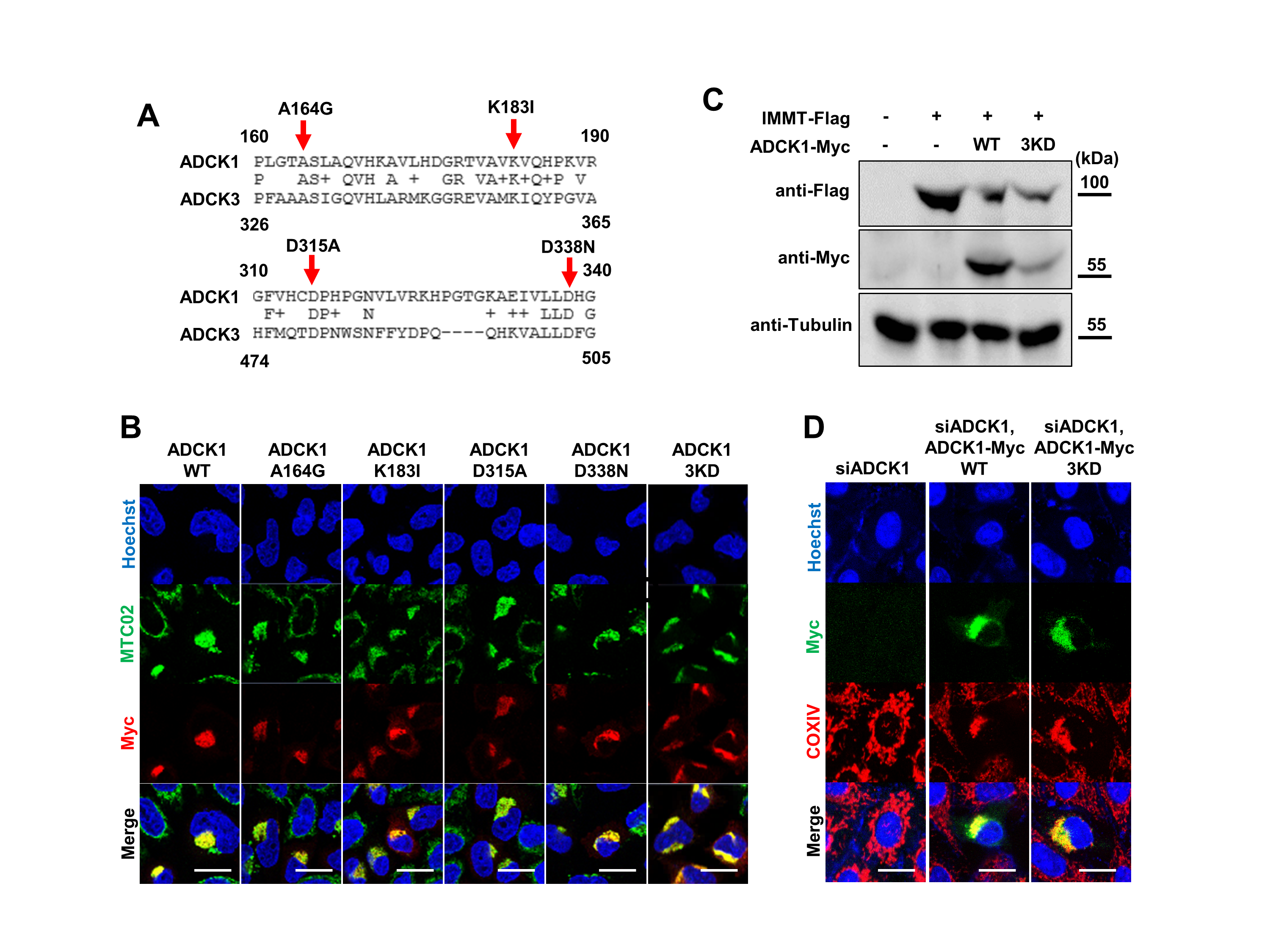

Supplement: S9 Fig — (A) A pairwise alignment of the kinase-related domains of ADCK1 and ADCK3. The arrows indicate the substituted positions to generate an ADCK1 kinase-dead form. (B) Fluorescent confocal images of the ADCK1 mutants in HeLa cells. HeLa cells were transfected with ADCK1 mutant constructs as indicated. The 3KD mutant contains triple mutations of K183I, D315A, and D338N. The Myc-tagged ADCK1 proteins were immunolabeled with anti-Myc antibody (red) and the mitochondria were labeled with anti-MTC02 antibody (green). Hoechst (blue) was used for nuclear staining. Scale bars, 20 μm. (C) HEK293T cells were transfected with IMMT-Flag and ADCK1-Myc (WT or 3KD) as indicated. WCL were prepared and analyzed for immunoblot with anti-Flag, anti-Myc and anti-tubulin antibodies. (D) Fluorescent confocal microscopy images of HeLa cells. HeLa cells were transfected with siADCK1 and ADCK1-Myc WT or ADCK1-Myc 3KD as indicated. The Myc-tagged ADCK1 proteins were immunolabeled with anti-Myc antibody (green) and the mitochondria were labeled with anti-COX IV antibody (red). Hoechst (blue) was used for nuclear staining. Scale bars, 20 μm. (TIF) [file pgen.1008184.s009.tif]

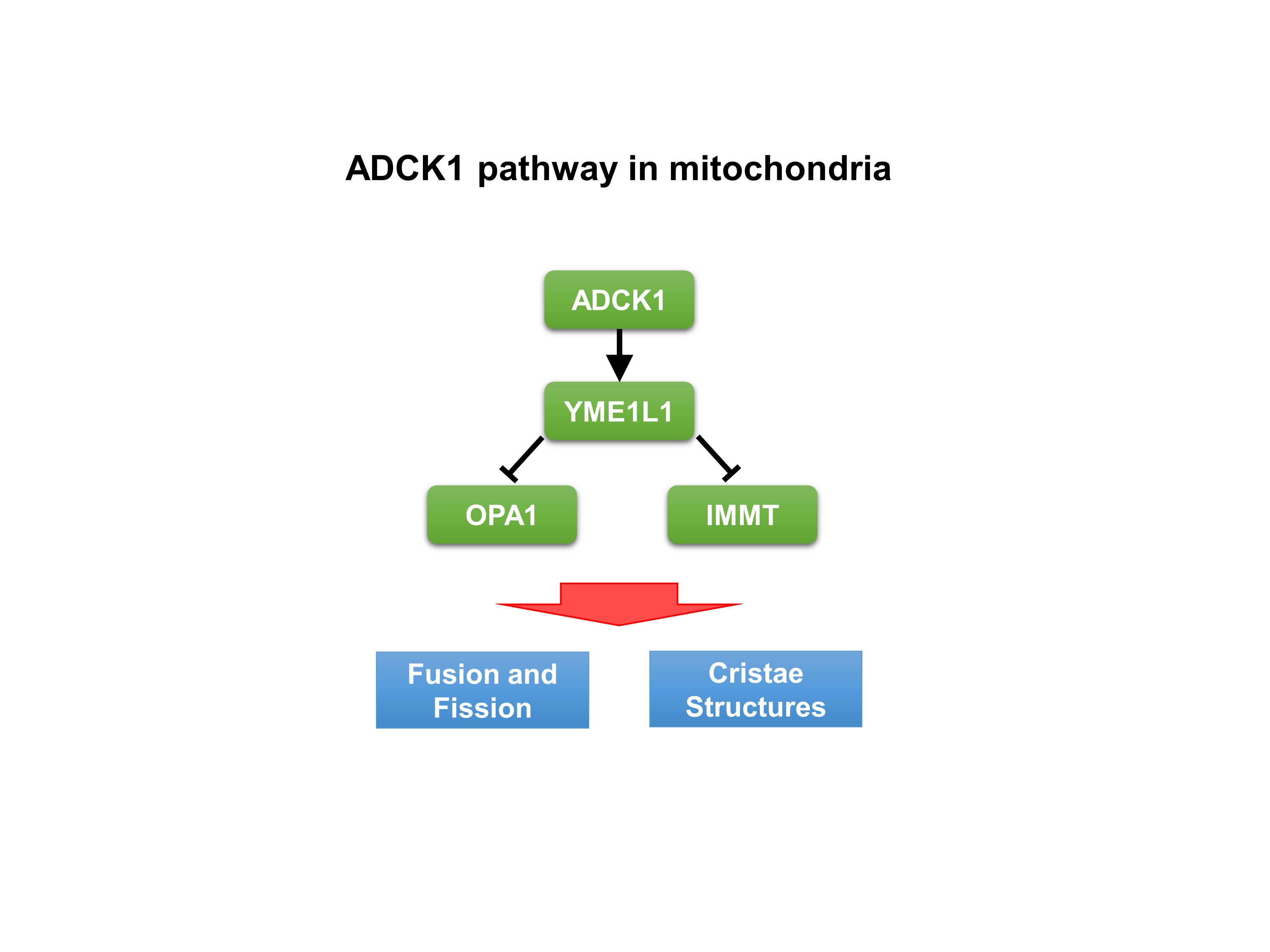

Supplement: S10 Fig — Dysfunctions in ADCK1 pathway induce mitochondrial cristae defects, mitochondrial fusion/fission imbalance, increased ROS, and apoptosis. (TIF) [file pgen.1008184.s010.tif]
